# Artificial Manganese Metalloenzymes with Laccase-like Activity: Design, Synthesis, and Characterization

**DOI:** 10.1021/acsabm.4c00571

**Published:** 2024-06-25

**Authors:** Carla Garcia-Sanz, Alicia Andreu, Mirosława Pawlyta, Ana Vukoičić, Ana Milivojević, Blanca de las Rivas, Dejan Bezbradica, Jose M. Palomo

**Affiliations:** † Instituto de Catálisis y Petroleoquímica (ICP), CSIC, c/Marie Curie 2, Campus UAM Cantoblanco, 28049 Madrid, Spain; ‡ Department of Microbial Biotechnology, Institute of Food Science, Technology and Nutrition (ICTAN-CSIC), José Antonio Novais 10, 28040 Madrid, Spain; § Faculty of Mechanical Technology, Silesian Technical University, Stanisława Konarskiego 18A, 44-100 Gliwice, Poland; ∥ Innovation Center of Faculty of Technology and Metallurgy, Karnegijeva 4, 11000 Belgrade, Serbia; ⊥ Faculty of Technology and Metallurgy, University of Belgrade, Karnegijeva 4, 11000 Belgrade, Serbia

**Keywords:** metalloenzymes, manganese, nanowires, artificial enzymes, laccase-like activity

## Abstract

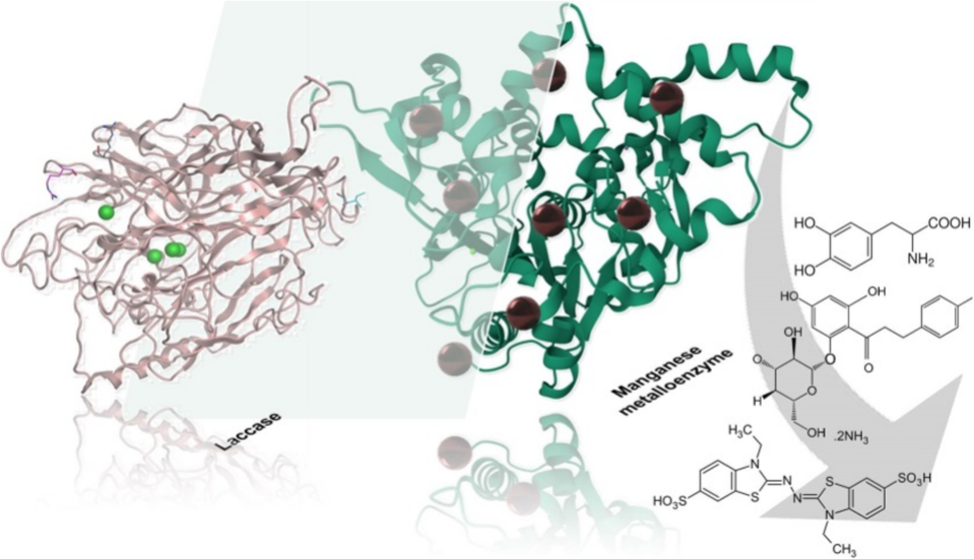

Laccase is an oxidase of great industrial interest due to its ability to catalyze oxidation processes of phenols and persistent organic pollutants. However, it is susceptible to denaturation at high temperatures, sensitive to pH, and unstable in the presence of high concentrations of solvents, which is a issue for industrial use. To solve this problem, this work develops the synthesis in an aqueous medium of a new Mn metalloenzyme with laccase oxidase mimetic catalytic activity. *Geobacillus thermocatenulatus* lipase (GTL) was used as a scaffold enzyme, mixed with a manganese salt at 50 °C in an aqueous medium. This leads to the in situ formation of manganese(IV) oxide nanowires that interact with the enzyme, yielding a GTL–Mn bionanohybrid. On the other hand, its oxidative activity was evaluated using the ABTS assay, obtaining a catalytic efficiency 300 times higher than that of *Trametes versicolor* laccase. This new Mn metalloenzyme was 2 times more stable at 40 °C, 3 times more stable in the presence of 10% acetonitrile, and 10 times more stable in 20% acetonitrile than Novozym 51003 laccase. Furthermore, the site-selective immobilized GTL–Mn showed a much higher stability than the soluble form. The oxidase-like activity of this Mn metalloenzyme was successfully demonstrated against other substrates, such as l-DOPA or phloridzin, in oligomerization reactions.

## Introduction

Laccase (EC 1.10.3.2, *p*-diphenol:dioxygen oxidoreductase) is an enzyme belonging to the group of blue copper oxidases.^1,2^ It contains oxidoreductases that couple the reduction of oxygen to water by four-electron reduction with the oxidation of a wide range of organic and inorganic substrates including phenols as well as some organic substances considered to be persistent organic pollutants (POPs), anilines, and aromatic thiols.^3−5^ Due to its broad substrate range and multiple roles, laccase has become a prominent research target in recent years. As a result, laccase has wide applications in biosensing and environmental remediation, as well as in the food, paper, and cosmetics industries.^6−9^ Indeed, in the last few years, one part of the research focused on enzymatic applications of laccases, whereas the other part focused on the production of novel polyphenolic compounds, which have been described as promising prebiotics—chemicals that are specifically used by their host microbes and have a favorable effect on the composition of the human microbiota.^10,11^

However, natural laccases have several drawbacks. The practical application of natural laccase is limited by its high cost, poor stability (pH, temperature, and storage time), difficulties in harsh environments, separation problems, and poor reusability.^12^ Immobilization of the enzyme usually increases its stability and reusability.^13^ This may be due to the interaction between the matrix and enzyme, which facilitates the stabilization of the peptide within the enzyme.^14^ However, enzyme activity on the support may be lost if the immobilization method alters the structure of the enzyme, and the search for a low-cost carrier that does not interfere with the action of the enzyme is still a problem.^15^ While there is a necessity to enhance the stability and recyclability of immobilized laccases, the ongoing exploration for new enzymes with heightened specificity for diverse applications is also underway. To overcome these shortcomings, efforts have been made to develop enzyme mimics. A particularly interesting area of research in recent years has been the synthesis of novel artificial metalloenzymes by combining metal or complex organometallic systems with enzymes. This area of study is growing both in terms of design and applications.^16^

Artificial metalloenzymes (ArMs) result from the incorporation of an abiotic metal cofactor within a protein scaffold.^17^ Four strategies have been reported for the synthesis of ArMs.^18^ The first approach focuses on Lewis base amino acids arranged in a cavity that can interact with a coordinative unsaturated metal (cofactor) through dative bonding. The second strategy is where the native metal of a metalloenzyme can be replaced by another metal, giving the protein new catalytic activity. The metal can be attached only to amino acids, as in the case of carboxypeptidase A, or it can be a member of a prosthetic group such as heme. The third is based on supramolecular interactions between a high-affinity inhibitor and a host protein, which can be used to bind a metal cofactor. The latter focuses on covalent immobilization, which can be achieved by irreversible interactions between complementary functional groups on the host protein or ligand.^19−21^ Significant progress has been made in the design and optimization of artificial metalloenzymes using these four anchoring techniques. As a result, it has been possible to generate metalloenzymes that are more selective and more active than those found in nature, as well as metalloenzymes with unique catalytic properties.^22,23^ Research in transition-metal manganese-based catalysts has recently increased due to their exceptional catalytic capability and multivalent nature.^24^ Mn-based nanomaterials are widely used in materials science, electronics, environmental protection, and biomedicine because of their ease of synthesis, low cost, environmental friendliness, and excellent physicochemical properties.^25−27^ Within this field, manganese oxide nanomaterials have been reported to have laccase-like activity.^28,29^ Manganese oxides (MnOx) are a major component of soils and sediments and occur naturally in over 30 different crystal forms. These forms are involved in several natural chemical processes. Certain MnOx are capable of oxidizing substrates by the transfer of a single electron, while the resulting reduced manganese oxides MnOx^red^ can be reoxidized to MnOx by dissolved oxygen that is reduced to water under certain conditions leading to a net result of electron shuttling from substrates to oxygen, like laccase.^30^ In addition, manganese oxides and laccase have comparable reactive capacities due to their common substrates, including ABTS (2,2′-azino-*bis* [3-ethylbenzothiazoline-6-sulfonic acid]-diammonium salt). Recently, a few examples have been described in the literature regarding manganese oxide catalysts with laccase-like activity.^31,32^ However, in most cases, they have large nanoparticle sizes and low stability. They also require complex synthesis conditions.

**Figure 1:**
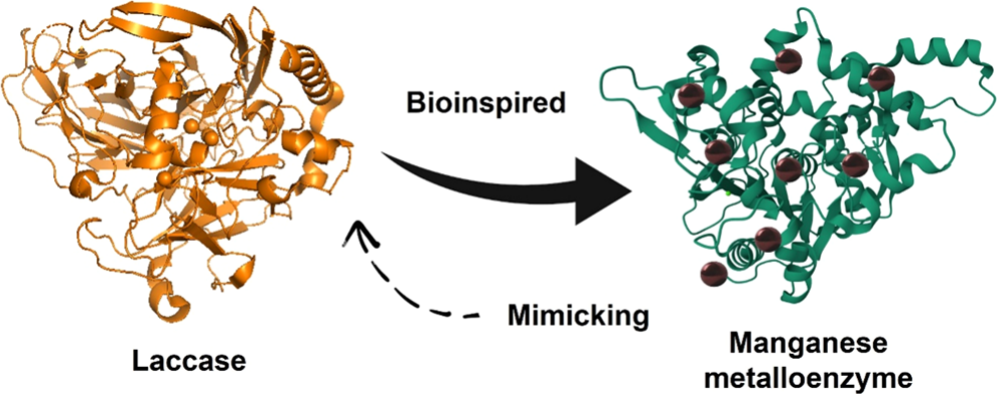
Conceptual model of the manganese metalloenzymes as laccase mimics proposed in this work.

Therefore, in this work, we describe a new strategy for the synthesis and design of artificial manganese metalloenzymes based on the in situ generation of manganese nanoparticles coordinated to the enzyme structure from manganese salts to create an enzyme–MnNPs bioconjugate with mimetic laccase-like activity (Figure 1).

This requires the selection of an enzyme type with a robust structure and characteristics that improve the stability and properties of the laccase. *Geobacillus thermocatenulatus* (GTL) is a thermo-alkalophilic lipase with high stability over a wide range of pH (9–11), temperature (50 °C), and organic solvents (2-propanol, acetone, methanol).^33^ GTL has two different lids (L1 and L2) in its structure, making it much more complex as it involves the movement and rearranging of 80 amino acids in the catalytic mechanism. In addition, this lipase has been reported to have high specificity for a wide range of substrates and high selectivity for resolving key intermediates in drug synthesis.^34^

These properties make this enzyme ideal for being used as a scaffold for the synthesis of the enzyme–MnNPs biohybrids. Thus, the hypothesis consists in the generation of in situ manganese nanoparticles induced by the enzyme, where they will be formed only on the protein, by a previous coordination step of manganese ions to the enzyme residues and then coalescence and final nanoparticle formation.^35−37^ The effects of the enzyme environment on the synthesis, morphology, and size of MnNPs were studied. Finally, the different artificial metalloenzymes were tested as catalysts in the oxidative processes.

## Experimental Section

### Chemicals

Prime Start HS Takara DNA polymerase was obtained from Takara Biotechnology (Jusatsu, Japan). PCR reactives were purchased from Applied Biosystems (MA). Primer synthesis was conducted by Fisher Scientific. Recombinant plasmid was sequenced by Secugen S.L. (Madrid, Spain). Restriction enzyme *Dpn*I was provided by Roche (Basel, Switzerland). Butyl-Sepharose 4 Fast Flow was from GE Healthcare (Uppsala, Sweden). *p*-Nitrophenylpropionate (*p*NPP) was obtained from Alfa Aesar (MA). 2,2′-Azino-*bis* [3-ethylbenzothiazoline-6-sulfonic acid]-diammonium salt (ABTS) was purchased from Thermo Scientific (MA). Triton X-100, dialysis tubing cellulose (avg. flat 33 mm), sodium citrate, sodium phosphate, l-DOPA, phloridzin, potassium permanganate (KMnO_4_), hydrogen peroxide (33%v/v), and laccase from *Trametes versicolor* were provided by Sigma-Aldrich (MA). Laccase from *Myceliophthora thermophila* expressed in *Aspergillus oryzae* (Novozym 51003) was from Novozymes (Bagsvaerd, Denmark). HPLC-grade acetonitrile was obtained from Scharlab (Barcelona, Spain).

### General Procedure for the Synthesis, Purification, and Characterization of Manganese Metalloenzymes in the Colloidal State

400 μL (70 μg protein) of the GTL enzyme solution (see the Supporting Information) was added to 3.6 mL of a solution containing 0.5 mg/mL (2000 equiv), 0.12 mg/mL (500 equiv), or 0.05 mg/mL (200 equiv) of the potassium permanganate salt (KMnO_4_) in distilled water. This solution was kept for 20 h under constant stirring at 130 rpm at 50 °C.

The manganese nanoparticle formation was followed by spectrophotometric measurement of the disappearance of the characteristic band of MnO_4_^–^ at 520–550 nm and the appearance of a peak at 367 nm corresponding to the formation of MnO_2_. After 20 h, the manganese metalloenzymes were obtained in the colloidal state. Their color varies from the initial purple to dark orange-brown depending on the added concentration of the KMnO_4_ salt.

Finally, a membrane with a molecular weight cutoff of 14 kDa was used for the dialysis purification of the manganese metalloenzymes. This technique makes it possible to purify the metalloenzyme by removing the permanganate salt remaining in the colloidal solution. They were kept under agitation for 4 h at room temperature in a beaker containing 1 L of distilled water, changing the water every 30 min (3 times). After this time, 1 mL of the purified manganese metalloenzymes (0.07 mg/mL) were obtained in the colloidal state. The synthesized manganese metalloenzymes were called **GTL@Mn2000eq**, **GTL@Mn500eq**, and **GTL@Mn200eq**.

### Preparation of Immobilized GTL–Mn Metalloenzymes

0.1 g of the immobilized enzyme (Bu–GTL)^34^ was added to 9 mL of a solution containing 0.5 mg/mL (2000 equiv), 0.12 mg/mL (500 equiv), or 0.05 mg/mL (200 equiv) of potassium permanganate salt (KMnO_4_) in distilled water. This solution was kept for 20 h under constant shaking at 130 rpm at 50 °C for 20 h. After this time, the solid of each sample was recovered by filtering this solution under vacuum, and then it was washed with distilled water (3 times, 20 mL) to obtain 0.1 g of the immobilized derivative. The synthesized manganese metalloenzymes were called **BuGTL@Mn2000eq, BuGTL@Mn500eq**, and **BuGTL@Mn200eq**.

### Fluorescence Spectroscopy Measurements

At room temperature, 3 mL of the corresponding colloidal Mn metalloenzyme was placed in a quartz cuvette with a path length of 1 cm, and the excitation–emission spectra of the free GTL and the synthesized manganese metalloenzymes were measured. The excitation wavelength was 280 nm, and the emission and excitation bandwidths were 5 nm. The fluorescence emission spectra were obtained between 200 and 500 nm.

### Gel Filtration of GTL–Mn Metalloenzymes

Gel filtration analyses were performed using a plastic column packed with beaded agarose-4BCL (column size 15 nm × 160 nm; column bed volume 8 mL). The eluting buffer was 10 mM sodium phosphate, pH 7.0; all separations were carried out at 25 °C with a flow rate of 1.2 mL/min, where 1 mL of **GTL@Mn2000eq** was added to the column and eluted with 15 mL of the indicated buffer. The eluate was collected in 0.5 mL aliquots and the laccase-like activity was determined by the ABTS assay at 420 nm. The molecular weight of **GTL@Mn2000eq** was estimated using standard proteins, laccase solution from *M. thermophila* expressed in *A. oryzae* (Novozym 51003) (85 kDa), Lipase B *Candida antarctica* (CALB) (33 KDa), and GTL containing 0.5% (v/v) Triton X-100 (43 kDa).

The activity of laccase was determined using the ABTS assay, while for CALB and GTL the enzymatic activity was determined using the *p*NPP assay at 348 nm.

### Evaluation of the Laccase-like Activity of the Manganese Metalloenzymes (ABTS Assay)

The ABTS assay was used to evaluate the laccase-like activity of the manganese metalloenzymes. To start the reaction, 5 μL of a 0.07 mg/mL dialyzed laccase solution from *M. thermophila* expressed in *A. oryzae* (Novozym 51003), 50 μL of a 0.07 mg/mL laccase solution from *T. versicolor*, or different amounts of the manganese metalloenzymes in the colloidal state, i.e., 5 μL (**GTL@Mn2000eq**), 15 μL (**GTL@Mn500eq**) or 50 μL (**GTL@Mn200eq**), or 50 μL of an emulsion of supported metalloenzymes (9 mg of the metalloenzyme supported and 500 μL of distilled water), were added under constant stirring to 2 mL of a standard 0.5 mM ABTS solution prepared in a 1:1 (v/v) ratio of 0.1 M sodium citrate buffer at pH 5 and 0.1 M sodium phosphate buffer at pH 5. After the addition of the Mn metalloenzyme, the solution changed from transparent to turquoise blue due to the formation of the radical species (ABTS^+**·**^). This color change was monitored by measuring the absorbance (λ = 420 nm) in the kinetic program at room temperature in a 1 cm path plastic cuvette.

To determine the laccase-like activity for each metalloenzyme, the ΔAbs/min value was calculated using the linear portion of the curve (ΔAbs). The specific activity (U/mg) was calculated using the following equation
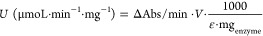
where the molar extinction coefficient (ε) of ABTS used was 36,000 M^–1^ cm^–1^ and mg _enzyme_ refers to mg of protein in the metalloenzyme.

### Stability of the Mn Metalloenzymes

The stability of different Mn metalloenzymes was evaluated by incubating them from 2 to 24 h at different temperatures (40 °C), different pHs (25 mM sodium phosphate at pH 4 and pH 8), or in the presence of acetonitrile as a cosolvent (10%, 20% (v/v)). Then, the laccase-like activity was used for monitoring the stability, considering the activity of artificial metalloenzymes at 25 °C in each case as the 100% value. The activity was determined by using the ABTS assay described above.

### l-DOPA Oxidation

In a 1 cm optical length plastic cuvette, 50 μL of **GTL@Mn2000eq** or free laccase (Novozym 51003) were added to 2 mL of a standard 1 mM solution of l-DOPA (3,4-dihydroxy-l-phenylalanine) in 0.1 M sodium phosphate buffer at pH 5 containing O_2_ (84 ppm). The solution changed from transparent to a reddish color due to the oxidation of l-DOPA to dopachrome. This color change was monitored by measuring its catalytic activity in a ultraviolet–visible (UV–vis) absorption spectrum at 475 nm in the kinetic program. An enzyme activity unit (U) was defined as the amount of enzyme causing an increase of absorbance by 0.001/min at 25 °C.^37^

### Synthesis of Phloridzin Oligomers

Oligomerization reactions were performed in 50 mL Erlenmeyer flasks on an orbital shaker at 150 rpm, in water, at a temperature of 40 °C. The total reaction mixture volume was 3 mL. Phloridzin concentration was 2 mg/mL, and the reaction was started using 60 mg of metalloenzyme. After 24 h, the reaction was stopped by removing the biocatalyst, **GTL@Mn500eq**. Control samples without enzymes were also prepared, and no products were detected. The samples were then analyzed on HPLC-UV.

### HPLC-UV and HPLC-MS Analysis of Oligomerization Products

For the analysis of the reaction mixture, reverse-phase high-performance liquid chromatography (HPLC) with UV–vis detection (Dionex UltiMate3000 HPLC system, Thermo Scientific) was used with Chromeleon 7.2 for data analysis. The analysis was performed with a ZORBAX Extend-C18 column (4.6 mm × 100 mm, particle diameter 3.5 μm, Agilent Technologies, Santa Clara) with a set temperature of 30 °C. As mobile phases, 0.1% (v/v) formic acid solution in deionized water (phase A) and acetonitrile (phase B) were used. A gradient elution was used as follows: 0–5 min 0–15% B, 5–35 min 15–40% B. The flow rate was 0.5 mL/min, and the detection wavelength was 280 nm. High-performance liquid chromatography–mass spectrometry (HPLC-MS) analysis of the starting monomer and the obtained reaction mixture was performed using a DionexUltiMate 3000 HPLC system (Thermo Scientific) coupled to a linear ion trap LTQ XL (Thermo Scientific). The previously described method for chromatographic separation on a ZORBAX Extend-C18 column was applied. Analytes were ionized using electrospray ionization (ESI) technique in the negative mode, forming deprotonated molecular ions. The optimal ion source parameters were as follows: source voltage (5 kV), sheath gas (18 au, i.e., eighteen arbitrary units), and capillary temperature (270 °C). Total ion chromatograms (TIC) were obtained by recording mass spectra in the range *m*/*z* 50–2000.

## Results and Discussion

### Synthesis and Characterization of Colloidal GTL@MnNPs Metalloenzymes

The first step was to produce and purify the GTL enzyme.^38−40^ For that purpose, the enzyme was initially adsorbed on a butyl-sepharose support (a technique that allows selectively reversible adsorption of lipases in the presence of other proteins).^38^ Then, once all of the lipase variants were absorbed (tested by enzyme activity), the immobilized derivative was treated in the presence of a buffered solution containing Triton X-100 (pH 7) to selectively desorb the variant and recover the enzyme in solution. This allows the GTL protein molecules to be obtained in an open form, stabilized by the detergent molecules (Figure S1).^33^

**Figure 2:**
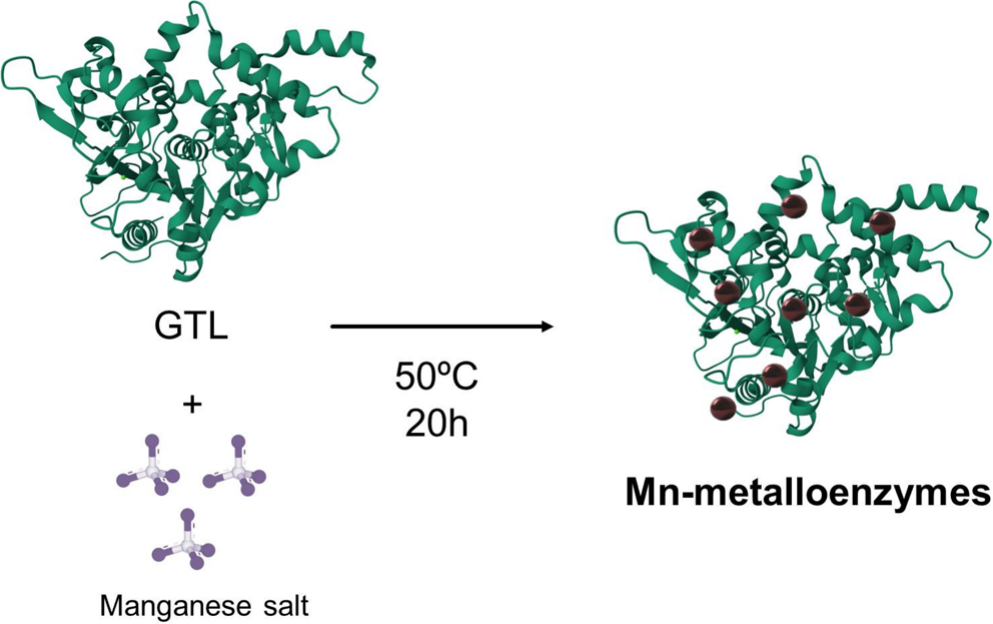
Schematic illustration of the synthesis of Mn metalloenzymes.

To prepare the artificial manganese metalloenzymes, various solutions of manganese salts (0.5 mg/mL) such as manganese(II) chloride tetrahydrate (MnCl_2_·4H_2_O), manganese(II) acetate tetrahydrate ((CH_3_COO)_2_ Mn·4H_2_O), manganese(II) sulfate monohydrate (MnSO_4_·H_2_O), and potassium permanganate (KMnO_4_) were prepared in distilled water and added to the previously obtained enzyme (Figure 2).

In the first attempt, the synthesis was performed at room temperature. However, no formation of MnNPs was observed at this temperature (data not shown). This can be explained by the thermophilic nature of the GTL enzyme (thermostable up to 45 °C). It therefore requires temperatures above 45 °C for activation. Thus, in the second approach, the solution was incubated with constant stirring at 50 °C. At this temperature, synthesis was only obtained when potassium permanganate was used in the presence of the enzyme, as indicated by a visible color change of the solution (from purple to orange), indicating the formation of MnNPs (Figure S2). No synthesis was observed for the other salts, as the solution remained clear (Figure S3). The permanganate salt was, therefore, the choice for the synthesis of the metalloenzymes.

Different concentrations of potassium permanganate (KMnO_4_) were prepared in distilled water and added to the desorbed GTL, giving three samples with different Mn equivalents: 2000 equiv (0.5 mg/mL), 500 equiv (0.12 mg/mL), and 200 equiv (0.05 mg/mL). The formation of metalloenzymes was followed by UV–vis spectroscopy by observing a steady decrease of all four absorption maxima corresponding to KMnO_4_ (506, 525, 545, and 566 nm) and the formation of a broad distinctive peak around 360–370 nm corresponding to the MnO_2_ nanoparticles.^41^ It is important to note that this color change is exclusively due to the reducing capacity of the enzyme, as it was not observed when the enzyme was not added (data not shown).

To understand the formation of metalloenzymes, the synthesis of metalloenzymes was studied during the first 20 min of incubation. Enzyme activity was measured using the pNPP assay, where most of the enzyme activity was lost. This may be related to the formation of MnNPs in the active site of the protein. To understand this, the fluorescence of the metalloenzyme was measured. A slight decrease in fluorescence was observed, due to the shielding of the tryptophan residues, as well as a shift toward the blue side of the spectra, indicating coordination of the enzyme with the metal (Mn). This was confirmed by powder X-ray diffraction (XRD), as the spectrum showed the initial formation of MnNPs, although the solution was still purple (Figure S4). The solution turned orange-reddish after 2 h, indicating the progress of the reduction reaction, and finally dark orange-brown (20 h), indicating the onset of the formation of MnO_2_ NPs^42^ (Figure S5). The maximum formation was obtained after 20 h incubation (Figure S6).

**Figure 3:**
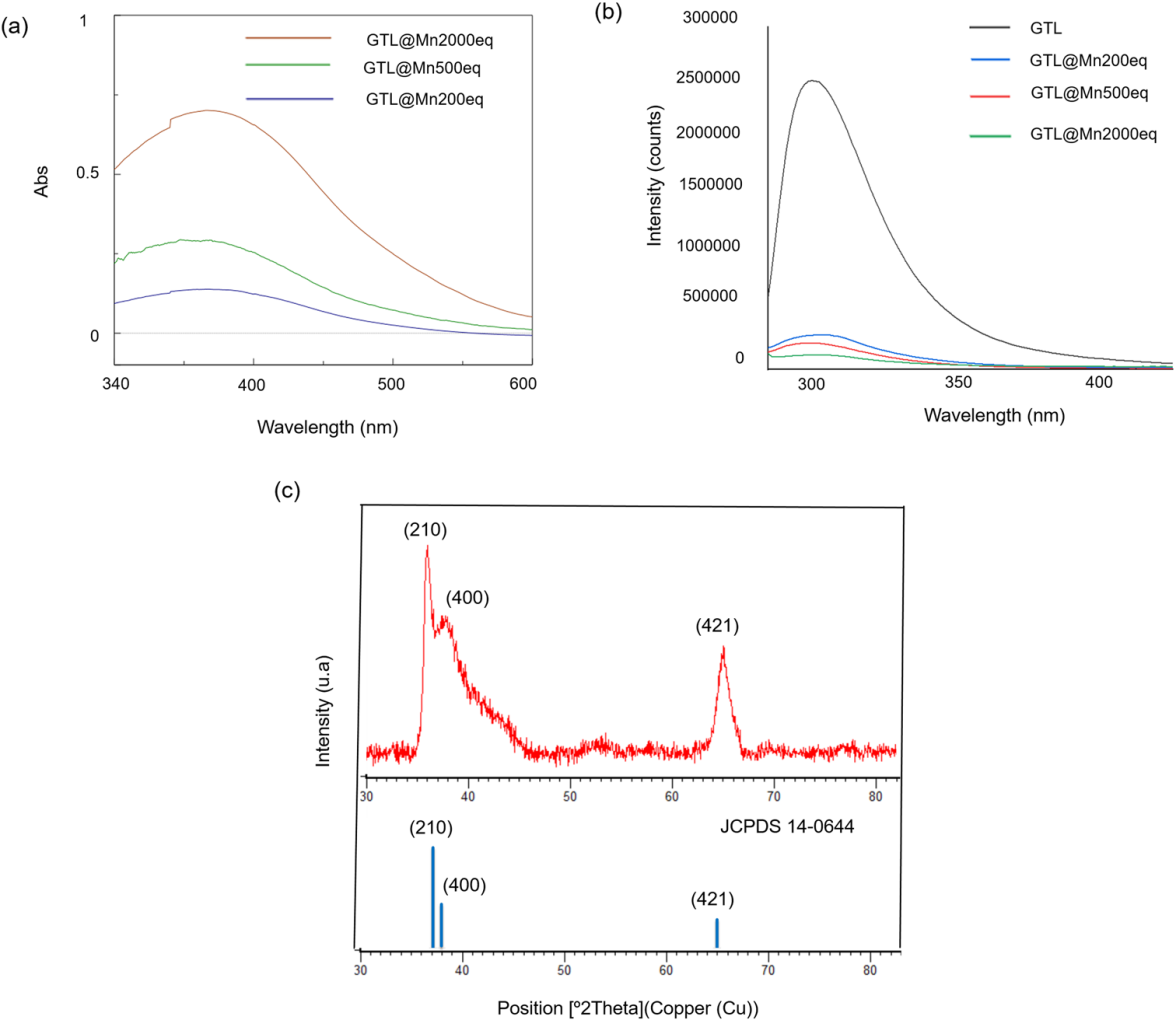
Characterization of the different manganese metalloenzymes. (a) UV-absorbance spectrophotometer spectra. (b) Fluorescence spectra (excitation wavelength 280 nm). (c) XRD pattern for **GTL@Mn2000eq**.

The highest Abs peak value was 0.7 for **GTL@Mn2000eq** at 367 nm when using 2000 equiv (Figure 3a). Lower concentrations of Mn (500 and 200 equiv) resulted in the formation of MnNPs with lower absorbance (0.29 and 0.13 Abs for **GTL@ Mn500eq** and **GTL@Mn200eq**, respectively) (Figure 3a). These results suggest the higher the absorbance, the higher the concentration of manganese in the bioconjugate. Furthermore, the coordination between the enzyme and the MnNPs is similar in all cases, as the peak wavelength does not shift between the samples. In addition, fluorescence analysis revealed that the three-dimensional structure of the protein changes as a result of the manganese coordination (Figure 3b) since the maximum of the native enzyme peak (303 nm) is slightly shifted toward the blue side of the spectrum by λ = 2 nm (301 nm) (Table S1). Near circular dichroism analysis also confirmed the effect on the protein structure by metal coordination (Figure S7). This has previously been reported with other modified proteins.^43^ In addition, higher Trp-quenching was observed for **GTL@Mn2000eq**, whereas **GTL@Mn200eq** presented a lower one. This shows that while the free conformation of lipase is more open, the conjugation with manganese modifies the three-dimensional structure, resulting in a decrease in fluorescence intensity and a shift in the conformation of the lipase—probably to a more closed form than the native one.^34^ Thus, higher concentrations of manganese (500 or 2000 equiv) lead to greater coordination of the metal with the protein and consequently to greater shielding of the tryptophan residues, which in turn reduces the signal. Further, increasing the equivalent of Mn (5000 equiv) resulted in saturation and precipitation of the enzyme (data not shown).

Next, powder X-ray diffraction (XRD) analysis was used to characterize the manganese structures generated in the conjugation with the protein (Figures 3c and S8). The XRD pattern shows the peaks for 2θ at 37° (210), 38.5° (400), and 65.4° (020) corresponding to the γ-MnO_2_ polymorph species in the sample (JCPDS 14–0644).^44^

**Figure 4:**
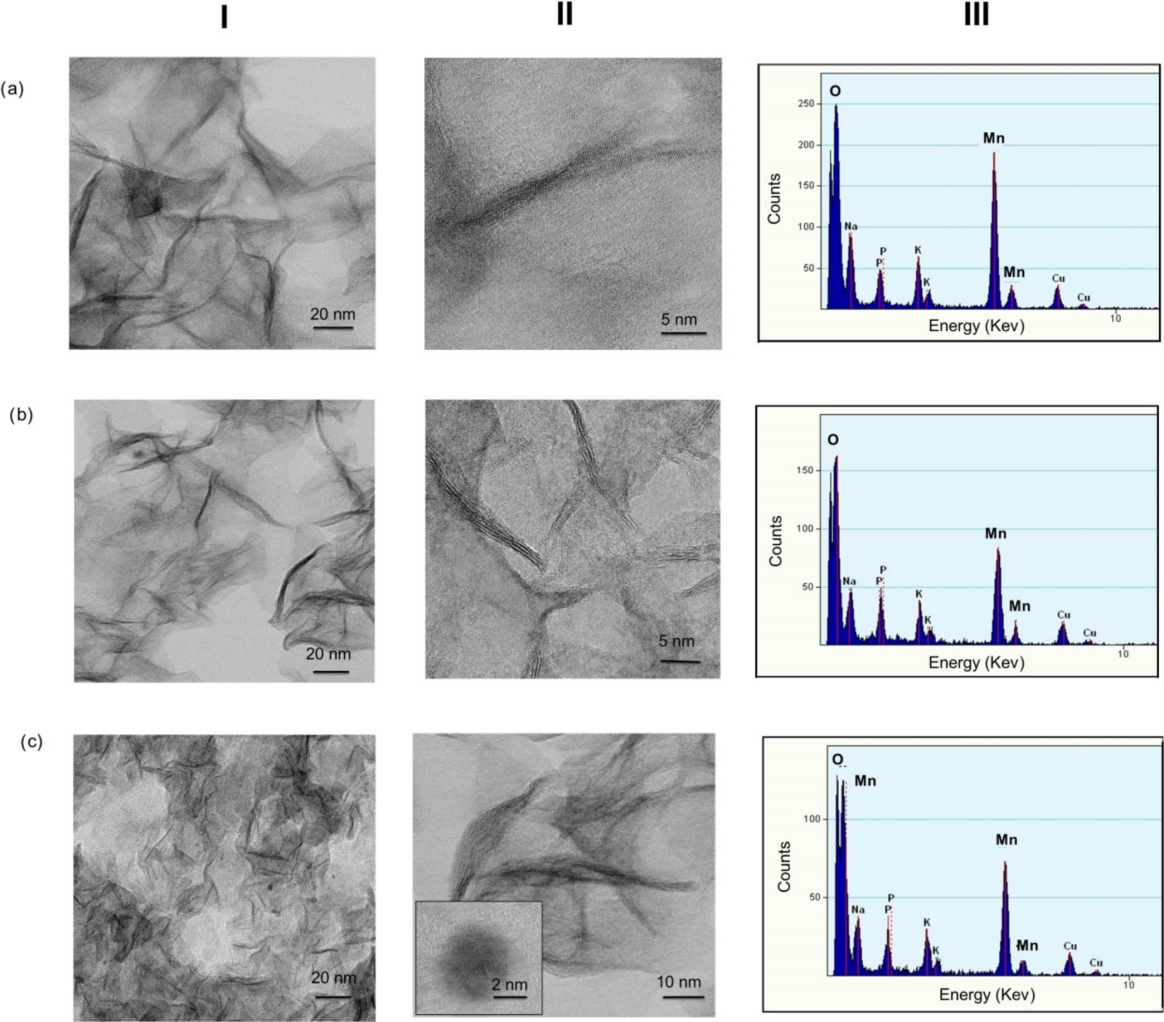
Characterization of manganese metalloenzymes (a) **GTL@Mn2000eq**, (b) **GTL@Mn500eq**, (c) **GTL@Mn200eq**: (I) transmission electron microscopy (TEM), (II) high-resolution TEM (HR-TEM), and (III) HAADF spectra.

High-resolution transmission electron microscopy (HR-TEM) revealed the formation of crystalline Mn nanowire structures (filaments of short length) in all of the colloidal GTL–Mn bioconjugates (Figures S9–S11). This morphology has been described as characteristic of the γ-MnO_2_ polymorph.^45^**GTL@Mn2000eq** showed the formation of nanowires (NWs) with a size of about 60 nm × 3 nm (Figure 4a), whereas smaller lengths were obtained for other manganese concentrations with an average size of 40 × 3 and 20 × 3 nm^2^ for **GTL@Mn500eq** (Figure 4b) and **GTL@Mn200eq** (Figure 4c), respectively. Therefore, these results suggest that the higher the concentration of manganese in the bioconjugate, the longer the length of the nanowires. Furthermore, these nanowires appear to be formed by the aggregation of spherical nanoparticles with an average diameter of 3.6 nm, as can be observed in **GTL@Mn2000eq** (Figure 4c,II).

The chemical composition of the nanoparticles was determined by high-annular dark-field imaging (HAADF) (Figure 4(III)). This technique confirms the presence of Mn and O in the sample, with the oxygen signal being twice as high as the manganese signal, indicating the presence of MnO_2_. The Cu peaks correspond to the signal detected by the TEM grid, while the P signal belongs to the buffer.

**Figure 5:**
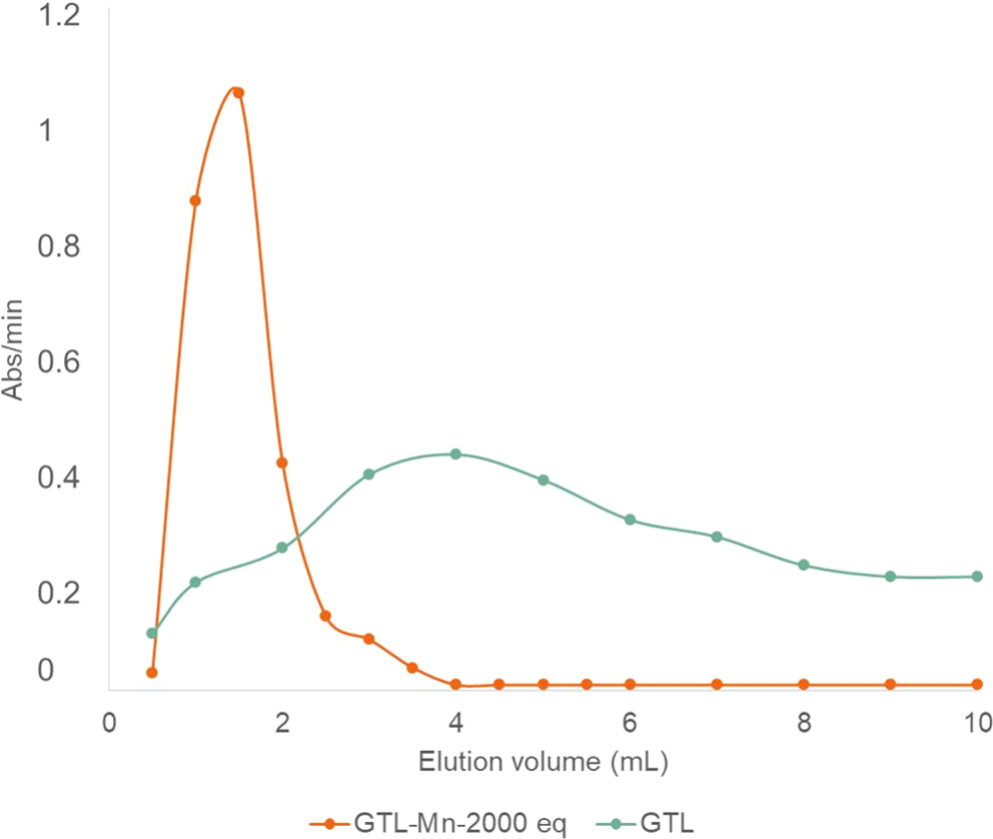
Elution profile in gel filtration chromatography of **GTL@Mn2000eq** (blue line) and GTL containing 0.5%(v/v) Triton X-100 (orange line).

It is well known that metal salts with lipases in homogeneous aqueous media have a strong tendency to form oligomeric structures.^46^ To see if this performance was conserved in the Mn metalloenzymes, **GTL@Mn2000eq** was evaluated by gel filtration chromatography (Figure 5). The eluent used in both cases was 10 mM phosphate buffer, pH 7. Under these conditions, only 43 kDa monomers were observed in GTL in the presence of 0.5% (v/v) Triton X-100. Interestingly, chemical modification with manganese is able to change the form of the native enzyme as a different elution profile was obtained.

The molecular weight of **GTL@Mn2000eq** was estimated from a calibration curve plotted using standard proteins (Figures S12–S14). The results showed the formation of a trimer with a molecular weight of 129 kDa.

Finally, the Mn content in the metalloenzymes was determined by inductively coupled plasma optical emission spectroscopy (ICP-OES) analysis. Results revealed a ratio of molecules of manganese per enzyme molecule of 345 in **GTL@Mn2000eq**, 112 in **GTL@Mn500eq**, and 29 in **GTL@Mn200eq** (Table S2).

**Figure 6:**
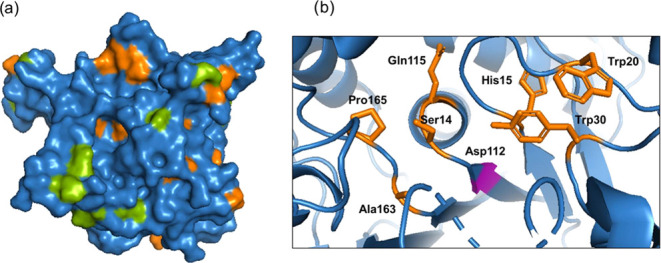
(a) Surface crystal structure of the active conformation of GTL, with marked aspartic acid (orange) and glutamic acid (green) residues. (b) Cartoon of the active site of GTL, with marked aspartic acid (pink) and environment residues (orange). The protein structure was obtained from the Protein Data Bank (PDB code: 2W22), and the picture was created using Pymol.

To understand the results obtained in the synthesis of Mn metalloenzymes, bioinformatics analysis of the enzyme structure was performed. In the coordination of the metal with the enzyme, Mn ions prefer to bind “hard” ligands such as oxygen of Asp and Glu.^47^ Sometimes, the N atoms of histidine can also bind Mn ligands in metalloenzymes.^47^ Synthesis took place at pH 5.5, meaning that in this case the imidazole ring of histidine is protonated (p*K*_a_ 6) and therefore MnNPs could bind to enzymes mainly through the carboxyl groups of glutamic and aspartic residues (Figure 6).

The manganese nanoparticles will bind to the protein’s most exposed carboxyl groups, conserving the negative charge free, not coordinated with neighboring NH^3+^ groups (e.g., arginine residues). The analysis of the crystallographic three-dimensional protein structure shows that Asp 366 (in the outer part of the oxyanion hole) seems to be the most probably accessible carboxylic group on the protein surface to be able to form the trimeric structure observed in Figure S15, with the different protein molecules being connected by the MnNPs coordination. This could also be linked to the loss of enzymatic activity as the nanoparticles block substrate access to the active site. Another aspartic acid is involved in the coordination sites for the rest of the MnNPs generated.

### Evaluation of the Laccase-like Activity of the Manganese Metalloenzymes

First, the laccase-like activity of the manganese metalloenzymes was evaluated in the selective oxidation of ABTS to ABTS^+·^. The reaction was carried out in an aqueous medium and at room temperature and compared with the free solution of the natural laccase from *T. versicolor* and laccase from *M. thermophila* expressed in *A. oryzae* (Novozym 51003).

**Figure 7:**
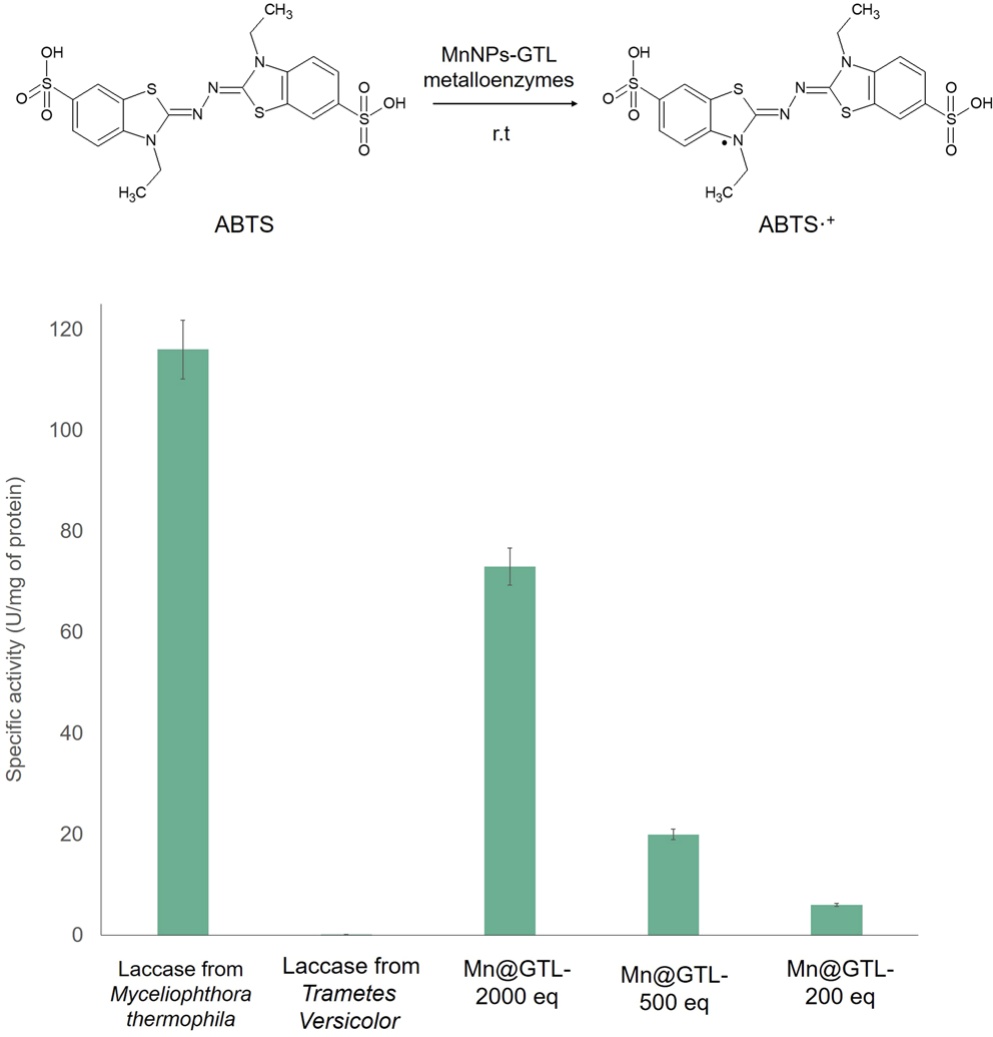
Laccase-like activity of the different Mn metalloenzymes at room temperature (rt) and pH 5.5 expressed in values of specific activity (U/mg of protein).

Figure 7 shows that the oxidative activity of the metalloenzymes increased with the Mn equivalents, with the highest specific activity being obtained for **GTL@Mn2000eq** (73 U/mg). This value was three times higher than the one obtained for **GTL@Mn-500 eq** (20 U/mg) and 12-fold higher than that of **GTL@Mn-200 eq** (6 U/mg). This high catalytic performance could be attributed to the presence of γ-MnO_2_ NPs in their structure, as it has been described in the literature that this manganese polymorph is the most potent in oxidizing ABTS.^31^

When the ABTS activity values of the Mn metalloenzymes are compared with those obtained for the native enzymes, it can be observed that **GTL@Mn2000eq** has half the specific activity of laccase from *M. thermophila* (116 U/mg) and more than 300 times greater activity than laccase from *T. versicolor* (0.21 U/mg). The high specificity of the 2000 equiv bioconjugate can be correlated with the presence of a higher longer number of γ-MnO_2_ NWs in the metalloenzyme structure, as has been reported in the literature to exhibit higher catalytic performance than the shorter ones.^48^

Thus, all of the Mn metalloenzymes showed laccase-like activity higher than that of the frequently used laccase from *T. versicolor*.

### Stability of the Colloidal Manganese Metalloenzymes

**Figure 8:**
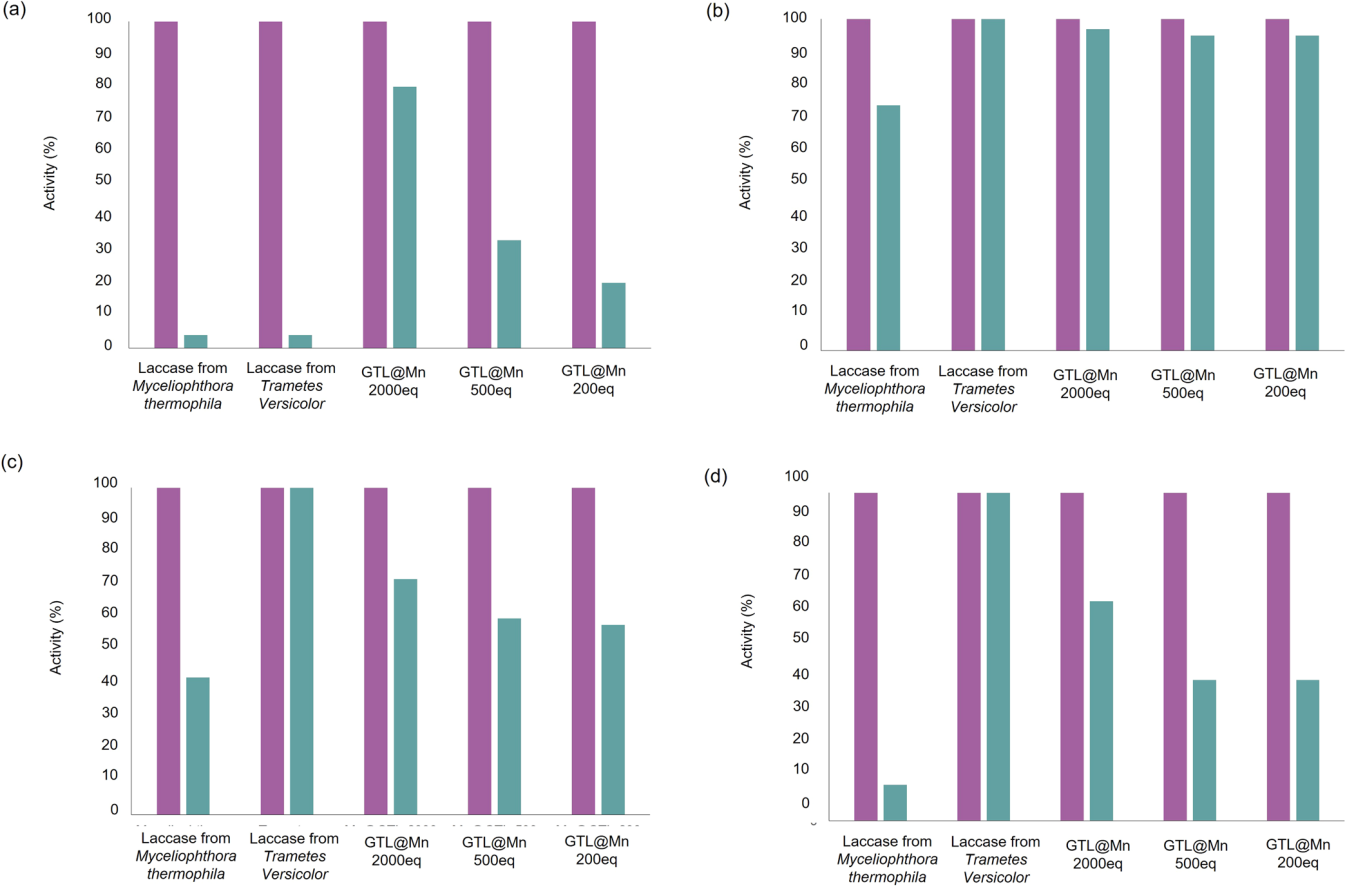
Stability of the different colloidal Mn metalloenzymes (GTL@Mn). (a) 40 °C, (b) sodium phosphate, pH 4 25 mM, (c) 10% (v/v) acetonitrile, and (d) 20% v/v acetonitrile. The purple column refers to the initial activity, and the green column refers to the activity after 2 h incubation.

Another important property of enzymes is their stability. In particular, laccase has been reported as not so stable under biological conditions.^49^ In this regard, the stability of the enzyme–Mn bioconjugates compared to laccase was evaluated at different temperatures and pH and in the presence of a cosolvent (Figure 8).

The different Mn–enzyme bioconjugates synthesized conserved up to 95% of their laccase-like activity at 40 °C, being nearly as stable as laccase from *T. versicolor* (Figure 8a). Interestingly, **GTL@Mn2000eq** was able to retain 80% of its activity when incubated at pH 4, being 20 times more stable than laccase from *M. thermophila* and laccase from *T. versicolor* (Figure 8b). However, it was not possible to measure the activity of the enzymes at pH 8 because they precipitated.

Furthermore, **GTL@Mn2000eq** turned out to be as stable as laccase from *T. versicolor* in the presence of 10% (v/v) acetonitrile and 2 times more stable than laccase from *M. thermophila*. When incubated with 20% (v/v) acetonitrile, this value increases up to 6 times. At these conditions, **GTL@Mn500eq** and **GTL@Mn200eq** retained approximately 60 and 40% of their activity values, respectively (Figure 8c–d).

Therefore, these data show that the use of GTL as a scaffold allows metalloenzymes to be more stable than natural laccases. This could be important in terms of maintaining the three-dimensional structure, which was also observed in the fluorescence experiments.

### Immobilized Mn Metalloenzymes

Lipases presented an extreme increase in stability when they are immobilized on hydrophobic supports, in particular, GTL in C4-functionalized macroporous support materials. This result is related to the fixing exclusively of the open conformation of the lipase on the support, which confers high stability of the enzyme against high temperature or the presence of cosolvent.^34^

**Figure 9:**
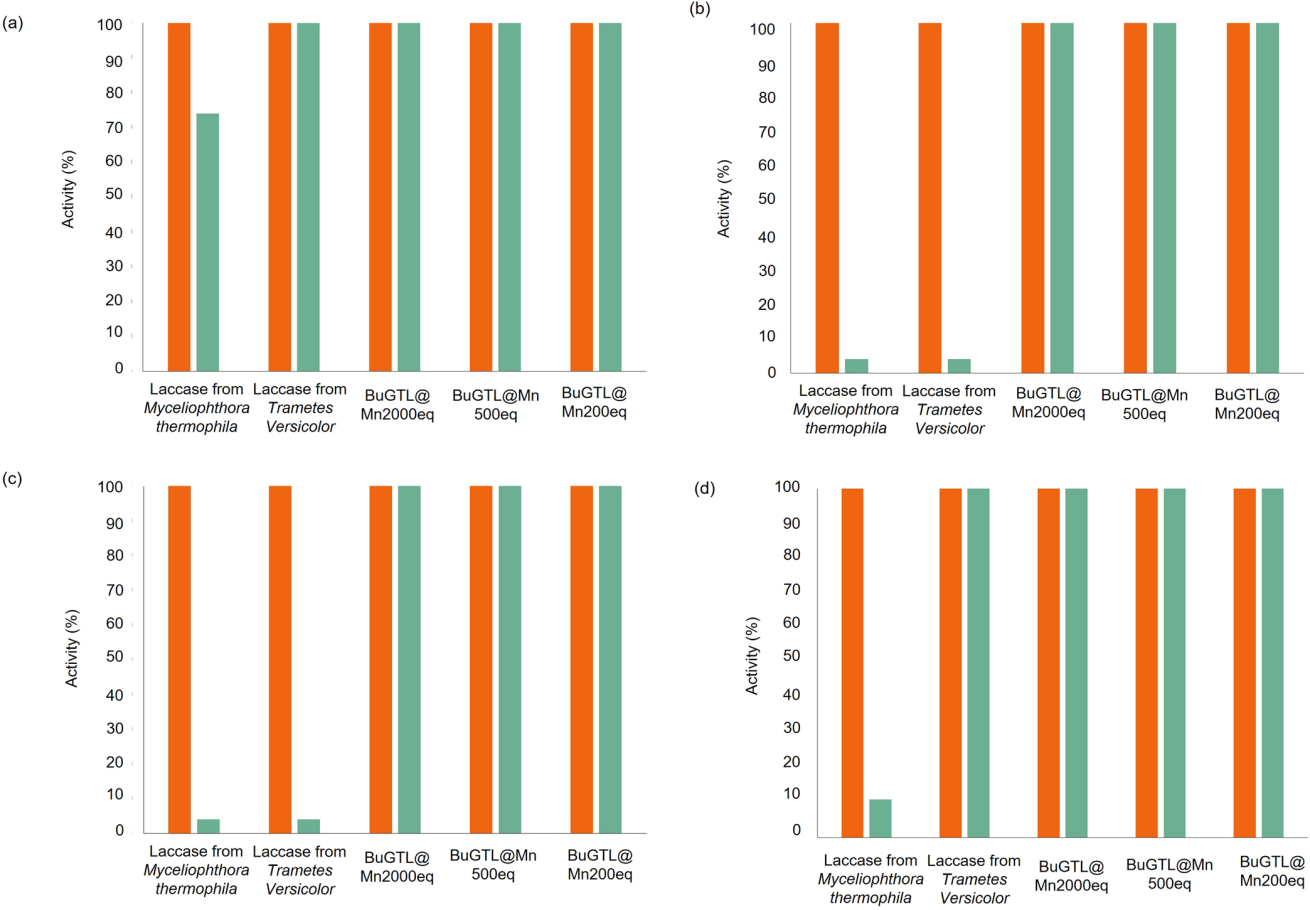
Stability of the different supported Mn metalloenzymes **(BuGTL@Mn)**. (a) 40 °C, (b) sodium phosphate pH 4 25 mM, (c) sodium phosphate, pH 8 25 mM, and (d) 20% v/v acetonitrile. The orange column refers to the initial activity, and the green column refers to the activity after 2 h incubation.

**Figure 10:**
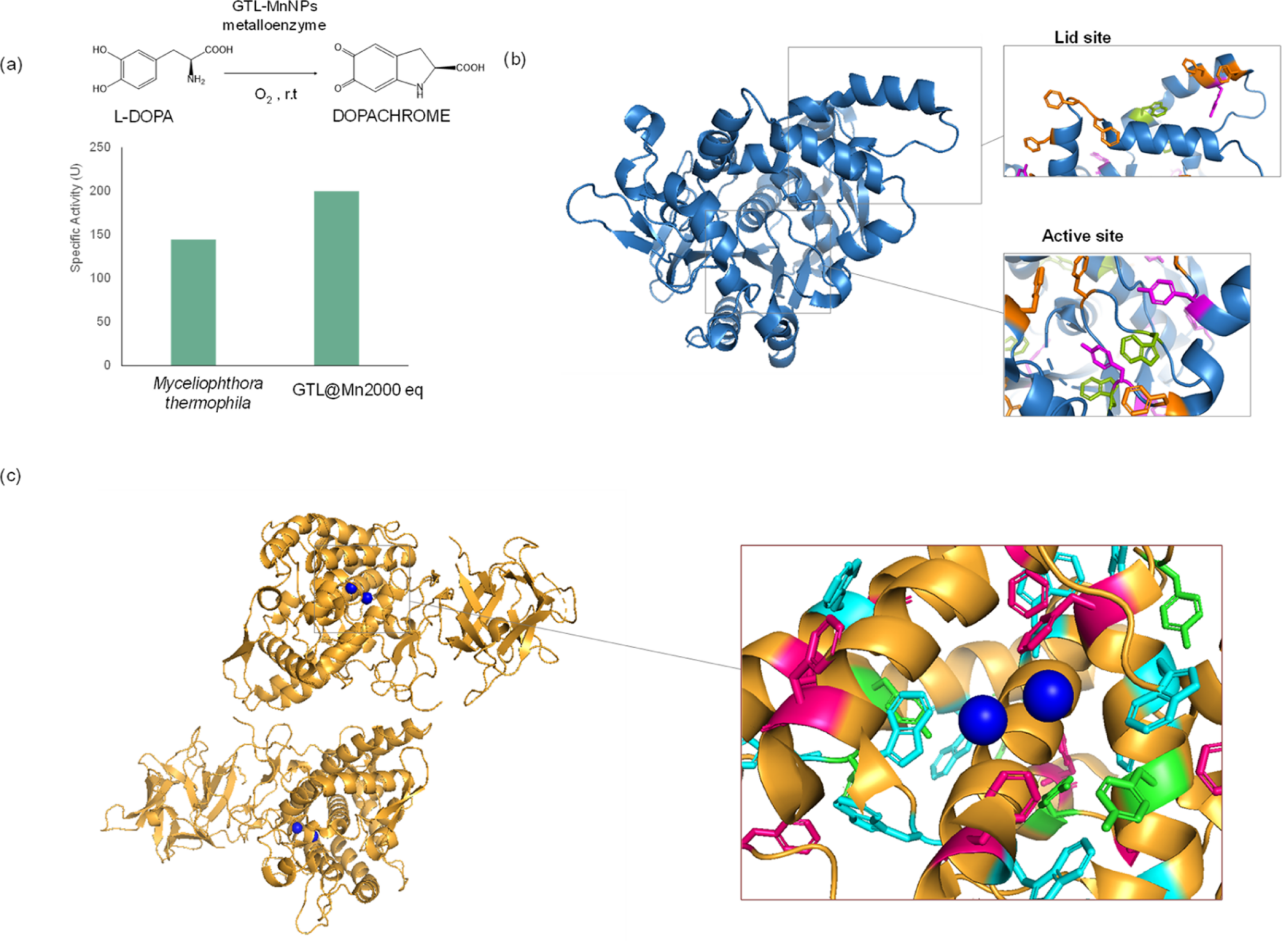
(a) l-DOPA oxidation reaction of laccase from *M. thermophila* and **GTL@Mn2000eq** in the presence of O_2_ (84 ppm) at rt and pH 5.5. (b) Cartoon of the crystallized GTL with marked tryptophan in green, phenylalanine in orange, and tyrosine residues in pink. (c) Cartoon of crystallized mushroom tyrosinase (TYR) with marked tryptophan in cyan, phenylalanine in pink, tyrosine residues in pink, and Cu atoms in blue. The protein structures were obtained from the Protein Data Bank (PDB code: 2W22 (GTL) and 2Y9W TYR), and the picture was created using Pymol.

Thus, in order to evaluate a potential industrial application of these artificial metalloenzymes and to increase their stability against different conditions, the new Mn metalloenzymes were prepared on the solid phase under similar conditions as soluble ones (Figures S16–S17). These immobilized metalloenzymes, named **BuGTL@Mn2000eq**, **BuGTL@Mn500eq**, and **BuGTL@Mn200eq**, were then characterized in terms of the Mn nanostructures formed (Figures S16–S17). TEM analysis showed the formation of nanowires as in the soluble metalloenzymes and also the formation of nanoparticles exclusively in the 2000 equiv one. The thermal and solvent stability of the immobilized Mn metalloenzymes was investigated (Figure 10). These data indicate that the supported metalloenzymes are at least 2 times more stable than the colloidal ones and as stable as laccase from *T. versicolor*, retaining 100% of their initial activity at high temperatures, much more stable over a wide pH range (4–8) and in the presence of cosolvent (20% v/v) after 2 h incubation (Figure 9). Therefore, the use of immobilization strategies improves the stability of the Mn–enzyme bioconjugates.

### Evaluation of the Oxidase-like Activity of the Manganese Metalloenzymes against l-DOPA

In order to evaluate the oxidase activity of the Mn metalloenzymes, l-3,4-dihydroxyphenylalanine (l-DOPA) reaction to dopachrome was performed in aqueous media and in the presence of O_2_ using natural laccase *M. thermophila* and **GTL@Mn2000eq**, which was found to be the most stable of the colloidal Mn–enzyme metalloenzymes (Figure 10a).

Laccase from *M. thermophila* presented a specific activity of 145 U. However, **GTL@Mn2000eq** exhibited a specific activity of 200 U, around 50% more active than the natural laccase. Therefore, this result again confirms the oxidase-like activity of the synthesized Mn metalloenzymes and indicates even higher activity in comparison with natural biocatalysts.

This activity may be related to its three-dimensional structure. GTL (Figure 10b) has a perfect trihistidine pocket near one of the two lids involved in the enzyme’s catalytic mechanism.^34^ This pocket is similar to that found in natural enzymes (e.g., mushroom tyrosinase). The two His pockets are surrounded by different amino acid residues (Trp, Phe, Tyr), which are important for substrate stabilization of the catechol group close to the Mn-binding position on the protein to allow the catalytic conversion, as occurs in the natural enzyme (Figure 10c).^50^

### Evaluation of the Oxidase-like Activity of the Manganese Metalloenzymes against Phloridzin Oligomerization

Finally, Mn–enzyme bioconjugate was tested in the structural modification of phloridzin in aqueous media at 40 °C (Figure S18). Supported Mn metalloenzyme (**BuGTL@Mn500eq**) was used for this purpose, as the immobilized metalloenzyme proved to be more stable than the colloidal bioconjugates. HPLC analysis of the reaction mixture revealed that, besides the peak of the starting monomer, two additional peaks that correspond to the products of the reaction of oligomerization appeared on the chromatogram. After 24 h of reaction, 20% of the starting monomer was converted into oligomers. Therefore, the reaction mixture was further analyzed by HPLC-MS. Figure S19 shows the chromatogram image where besides phloridzin (*m*/*z* 435, retention time 14 min), two dimer (*m*/*z* 869) molecules at retention times 21 and 21.5 min, respectively, appeared in the mixture. Extinction coefficients of dimers are significantly lower in comparison with extinction coefficients of phloridzin; hence, they appear even smaller in chromatograms. After analyzing the molecular weights of the identified products, it could be seen that during the formation of the connection between the phloridzin units, the loss of two hydrogen atoms occurs.

## Conclusions

This work shows the successful development of a novel method for the synthesis of a new type of artificial manganese metalloenzyme using *G. thermocatenulatus* lipase as a three-dimensional scaffold. In all cases, the formation of nanoparticles in the protein matrix was induced in situ by the enzyme without the use of an external reducing agent. The final nanostructure formation of MnO_2_ species is influenced by the experimental conditions, thereby promoting the formation of nanowires within the protein matrix. All of these enzyme–Mn bioconjugates showed high specificity in the ABTS assay with mimetic laccase-like activity, in particular, **GTL@Mn2000eq**, which was more than 300 times that of *T. versicolor* laccase. In addition, the use of immobilization strategies improves the stability of these artificial metalloenzymes. Immobilized biocatalysts were highly stable at 40 °C, in a wide range of pH (4–8), and in the presence of cosolvent (acetonitrile 20% v/v). The metalloenzymes also showed oxidative capacity in other reactions such as l-DOPA oxidation and phloridzin oligomerization. This technology allows the transformation of lipase into an oxidase.

The advantages of the development of artificial metalloenzymes versus natural laccase are as follows: (i) Highest accessibility, for example, using an overexpressed protein as a protein scaffold resulting in the cheapest final biotechnological process. (ii) Highest stability, especially when the scaffold protein used is a thermostable enzyme. This allows the enzyme to be used over a wide range of pH or T where, for example, laccase from *T. versicolor* is not able to operate. (iii) The simplicity and sustainability of this methodology to create metalloenzymes in aqueous media and at room temperature compared to other strategies where, for example, one is able to synthesize an organometallic compound beforehand, with subsequent conjugation.

Future research will aim to expand the use of metalloenzymes in different oxidation reactions, optimizing and expanding the applicability in oligomerization reactions of active molecules focused on promising products for the food and cosmetic industry.

## Supporting Information Available

Additional experimental details (characterization methods, expression, production, and purification of *G. thermocatenulatus* lipase), figures of enzyme crystal structure (Figure S1 and S15), additional data of synthetic procedure of artificial Mn enzymes (Figures S2, S3, S5, and S6), characterization of the new artificial metalloenzymes (TEM, XRD) (Figures S4 and S7–S11), gel filtration chromatography experiments (Figures S12–S14), characterization of immobilized Mn enzymes (Figures S16 and S17), oligomerization process (Figures S18 and S19), fluorescence data for the manganese metalloenzymes (Table S1), and protein and Mn content of the manganese metalloenzymes (Table S2) (PDF)
