# Supplementary material for: Artificial Manganese Metalloenzymes with Laccase-like Activity: Design, Synthesis, and Characterization
Source: ACS Appl Bio Mater. 2024 Jun 25;7(7):4760–71. doi: 10.1021/acsabm.4c00571 (PMC11253090; doi:10.1021/acsabm.4c00571)
Supplement: Supplementary file 1 — mt4c00571_si_001.pdf [file mt4c00571_si_001.pdf]

Supplementary information

Artificial manganese metalloenzymes with laccase-like activity: Design, Synthesis and Characterization

Carla Garcia-Sanz[1], Alicia Andreu[1], Mirosława Pawlyta[3], Ana Vukoičić [4], Ana Milivojević [5], Blanca de las Rivas[2], Dejan Bezbradica[5], and Jose M. Palomo*, [1]

[1]Instituto de Catálisis y Petroleoquímica (ICP), CSIC, c/Marie Curie 2, Campus UAM Cantoblanco, 28049 Madrid (Spain)

[2] Department of Microbial Biotechnology, Institute of Food Science, Technology and Nutrition (ICTAN-CSIC), José Antonio Novais 10, 28040 Madrid, Spain

[3]Faculty of Mechanical Technology, Silesian Technical University, Stanisława Konarskiego 18A, 44-100 Gliwice, Poland

[4] Innovation Center of Faculty of Technology and Metallurgy, Karnegijeva 4, 11000 Belgrade, Serbia

[5] Faculty of Technology and Metallurgy, University of Belgrade, Karnegijeva 4, 11000 Belgrade, Serbia

*Correspondence: josempalomo@icp.csic.es

Experimental part

Characterization methods

PCR amplifications were performed in a *Personal Mastercycler gradient* (Eppendorf, Gemany) thermocycler. Isolation plasmids were carried out using QIAquick Gel extraction (QIAGEN, Germany). Spectrophotometric analyses were run on a V-730 spectrophotometer (JASCO, Tokyo, Japan). Inductively coupled plasma-optical emission spectroscopy (ICP-OES) was performed of the solid material. 100 μL or 10 mg of the solid powder or was treated with 6 mL of HCl (37% v/v) for digestion. Then, it was added with 9 mL of water, centrifuged, and the clear solution was analysed for Mn content. Inductively Coupled Plasma-Optical Emission Spectrometry (ICP-OES) was performed on an OPTIMA 2100 DV instrument (PerkinElmer, Waltham, MA, USA). X-Ray diffraction (XRD) patterns were obtained using a Texture Analysis D8 Advance Diffractometer (Bruker, Billerica, MA, USA) with Cu Kα radiation. Fluorimetric measurements were performed in a Fluoromax Plus fluorimeter (Horiba, Scientific, Tokyo, Japan). Mn nanoparticles sizes and morphology were determined by transmission electron microscopy (TEM). Images were obtained using an an S/TEM Titan 80-300 microscope equipped with a Cetcor Cs probe corrector and energy dispersion X-ray spectrometer (EDS) for chemical composition analysis. Samples for TEM observation were prepared by dispersing a small amount of the material in ethanol and putting a droplet of the suspension on a microscope copper grid covered with carbon film and allowed to evaporate the alcohol. Then, samples were dried and purified in a plasma cleaner. TEM (Bright Field BF, Dark Field DF, and Selected Area Diffraction) and STEM modes (BF detector to show the structure and morphology; and High Angle Angular Dark Field HAADF detector to reveal chemical contrast (Z-contrast)) were used for imaging. Because the tested material was sensitive to the electron beam, during

microscopic observations, the intensity of the electron beam and the exposure time were limited. Chromatographic analyses were run at 25 ºC using an HPLC pump PU-4180 (JASCO, Tokyo, Japan) and an UV-4075 UV-Vis detector (JASCO, Tokyo, Japan).

Expression and production of *Geobacillus thermocatenulatus* lipase (GTL)

The gene corresponding to the mature lipase from *G. thermocatenulatus* (GTL, formerly BTL) was previously cloned into the pT1 expression vector. The gene encoding the Cys65Ser/Cys296Ser GTL mutant (GTLmutCys) was constructed using mutagenic primers by site-directed mutagenesis PCR. The amplification reaction was performed using 2 µL of previously purified plasmid pT1BTL2 as a template, in a final volume of 50 µL, under the appropriate conditions for Prime Start HS DNA Polymerase according to the supplier's instructions, using an extension time sufficient to copy the entire template vector (6 min). First, the Cys65Ser mutant was constructed using primers Cys65Ser-for (CAACTGGGACCGGGCGAGCGAAGCGTACGCCCAG) and Cys65Ser-rev (CTGGGGTACGCTTCGCTCGCCCGGTCCCAGTTG) for the Cys65Ser change. The PCR product was digested with the DpnI endonuclease, which restricts only methylated DNA, to eliminate the template plasmid. *E. coli* DH10B cells were transformed directly with the digestion product (10 µL) using competent cells previously obtained by the RbCl method. Recombinant plasmids containing the expected nucleotide changes were isolated and identified by sequencing. Once the Cys65Ser mutant was obtained, the double mutant was constructed in a similar way, using the primers Cys296Ser-for (GCGGTCGTAAGCGCCCCGTTTC) and Cys296Ser-for (GAAACGGGCGCTTACGACCGC) for the Cys296Ser change and the Cys65Ser construction as template. Again, the PCR product was digested with DpnI and *E. coli* DH10B cells were transformed with the digest. Finally, the recombinant plasmid with the de double mutation (pT1GTLmutCys) was identified by sequencing. *E. coli* DH10B cells

carrying the recombinant pT1GTLmutCys plasmid (1L) were grown in Luria-Bertani medium containing ampicillin (100 μg/ml) at 30°C and overexpression was induced by raising the temperature to 42°C for 20 hours. Cells were then harvested by centrifugation at 4000 rpm for 15 minutes. The cell pellet was resuspended in 20 mL of phosphate buffer 50 mM pH 7 containing 300 mM of NaCl and then disrupted by French press passages (3 times at 1100 psi). The insoluble fraction of the lysate was removed by centrifugation in an SS34 rotor at 20000 rpm for 30 min at 4°C using a Sorvall centrifuge and the supernatant was filtered through a 0.45μm pore filter.

Purification of GTL enzyme

10 g of the butyl-Sepharose support (Bu) were incubated in a plastic container with 10 mL of the corresponding enzyme (70 mg) and 190 mL of 25 mM sodium phosphate buffer pH 7 for 24 hours. To verify that the enzymes were adsorbing onto the support, the decrease in enzyme activity of the supernatants was measured at different times using the *p*NPP enzyme activity assay. This was done by measuring the absorbance (λ=348 nm) produced by the release of p-nitrophenol (*p*NP) on the *p*NPP hydrolysis (50 mM) in sodium phosphate buffer (25 mM, pH 7). To initiate the reaction, 20 µL of *p*NPP standard solution (prepared in acetonitrile) was added to 2.5 mL of phosphate buffer. Then, 20 µL of the supernatant were added under magnetic stirring. After 24 hours, the enzyme activity is zero as all the enzyme is adsorbed on the support (Butyl-GTL). Then, the solid was washed with plenty of water and finally GTL enzyme was desorbed from the support by re-suspending the immobilised enzyme in a 1:10 (w/v) ratio in sodium phosphate buffer (25 mM pH 7) containing 0.5%(v/v) Triton X-100. This mixture was incubated for 1 hour at room temperature. After that, the support was removed by centrifugation and the soluble enzyme was obtained in the supernatant.

Figures

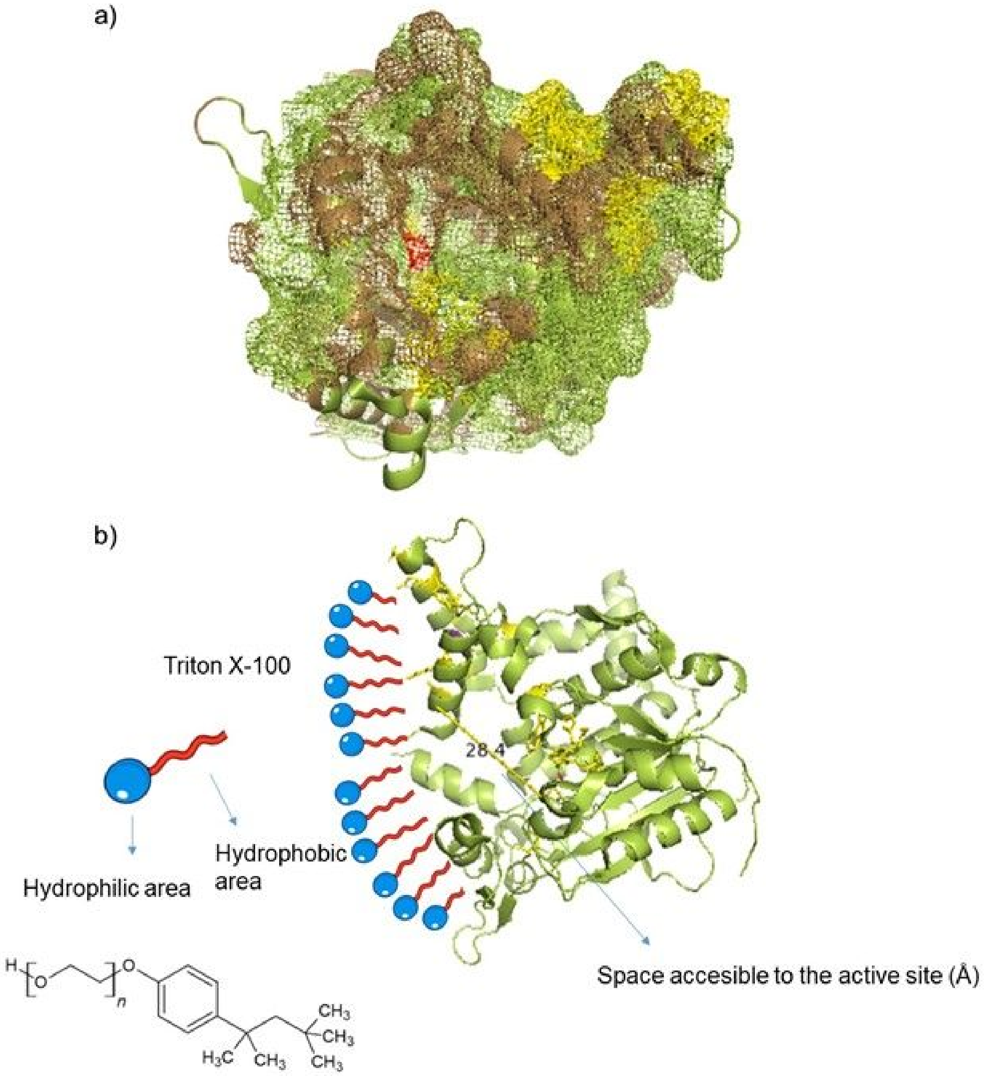

Fig. S1. (a) Crystal structure cartoon of the open conformation of GTL, marked in yellow lid site and in brown hydrophobic residues. (b) Representation of the stabilization of open conformation of GTL by triton X-100 molecules v*ia* hydrophobic interactions. The protein structure was obtained from the Protein Data Bank (pdb code: 2W22) and the picture was created using Pymol.

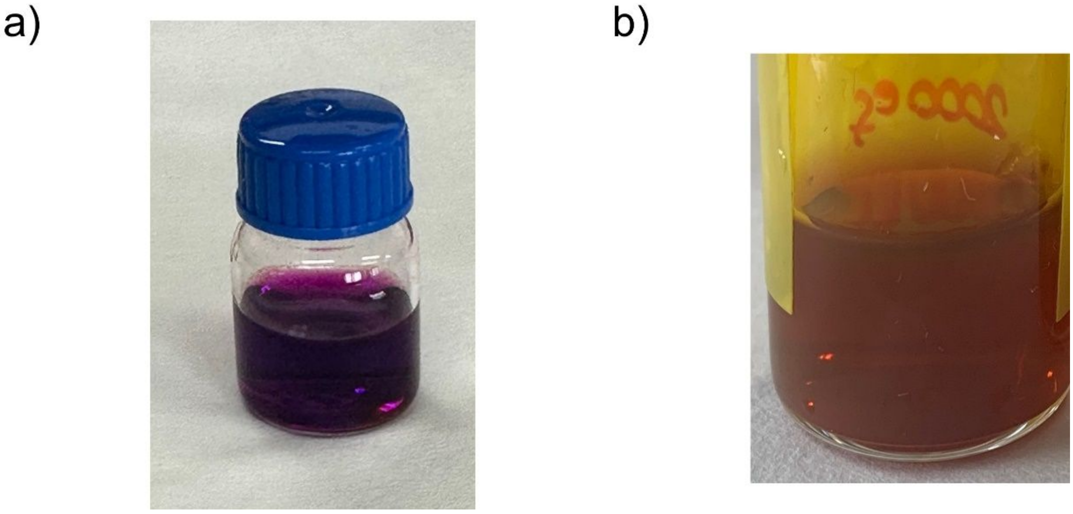

Fig. S2. (a) Initial permanganate solution (b) Solution after 20 h incubation at 50ºC.

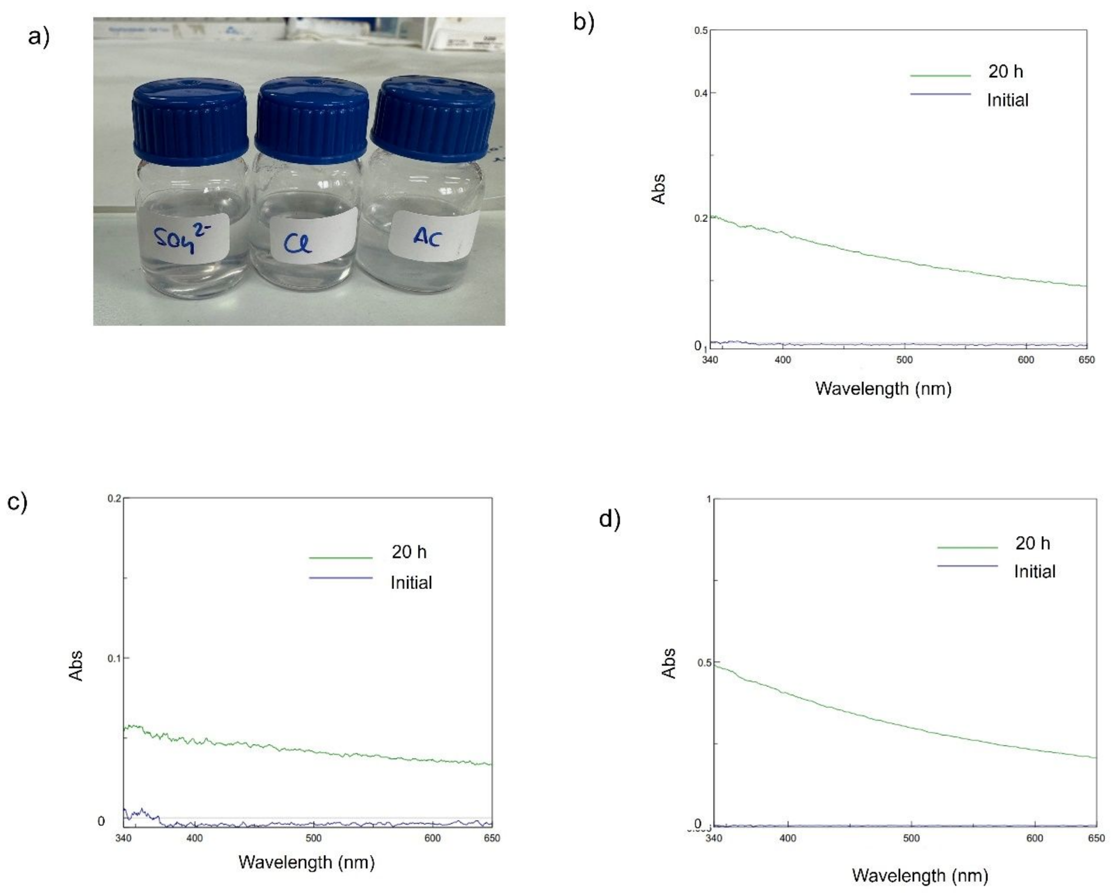

Fig. S3. (a) Manganese solutions after 20 h incubation at 50ºC for manganese sulphate, manganese chloride and manganese acetate. (b) Synthesis profile for manganese sulphate salt. (c) Synthesis profile for manganese acetate salt. (d) Synthesis profile for manganese chloride salt.

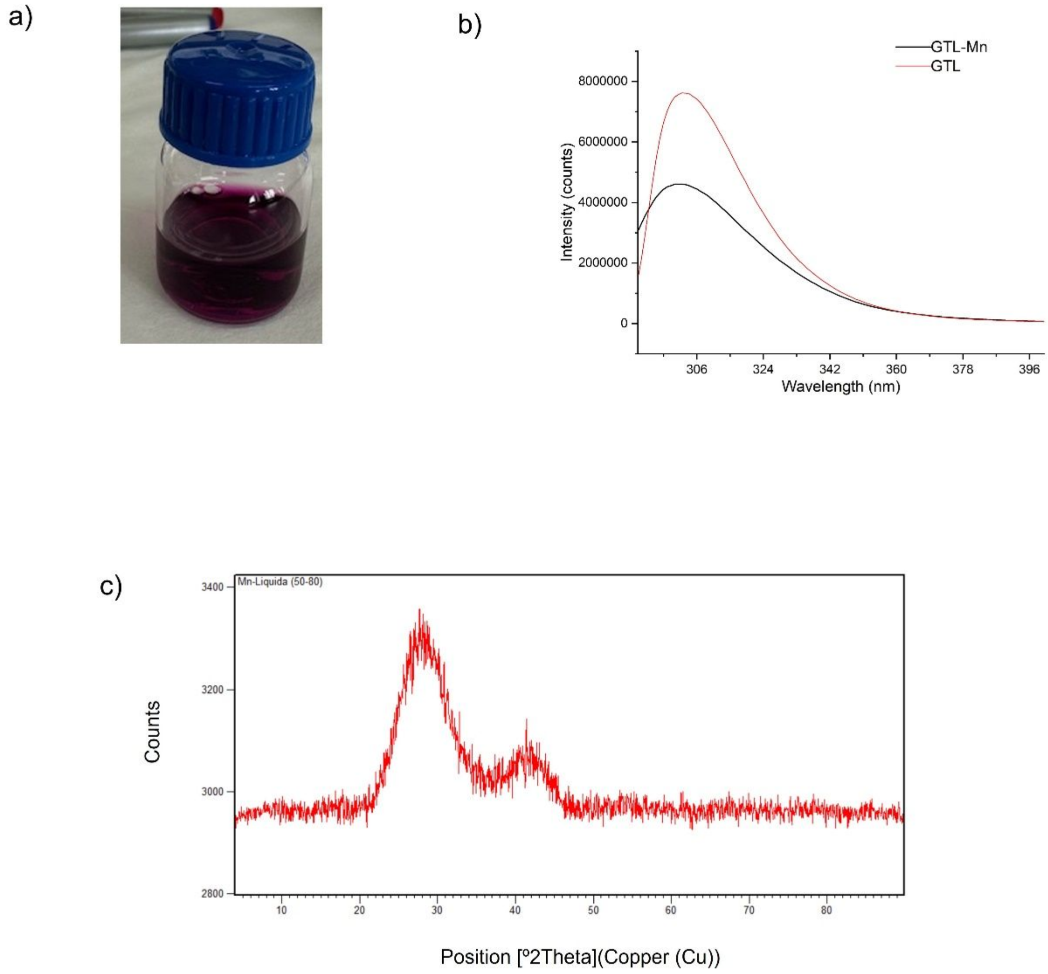

Fig. S4. Characterization of **GTL@Mn2000 eq** after 20 minutes of incubation (a) Solution after 20 minutes of incubation at 50ºC. (b) Fluorescence spectra (excitation wavelength 280 nm) (c) XRD pattern.

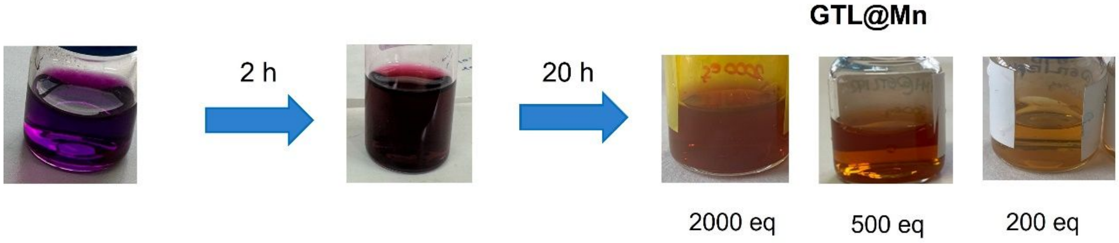

Fig. S5. Initial solution, after 2 h and 20 h of the synthesised manganese metalloenzymes.

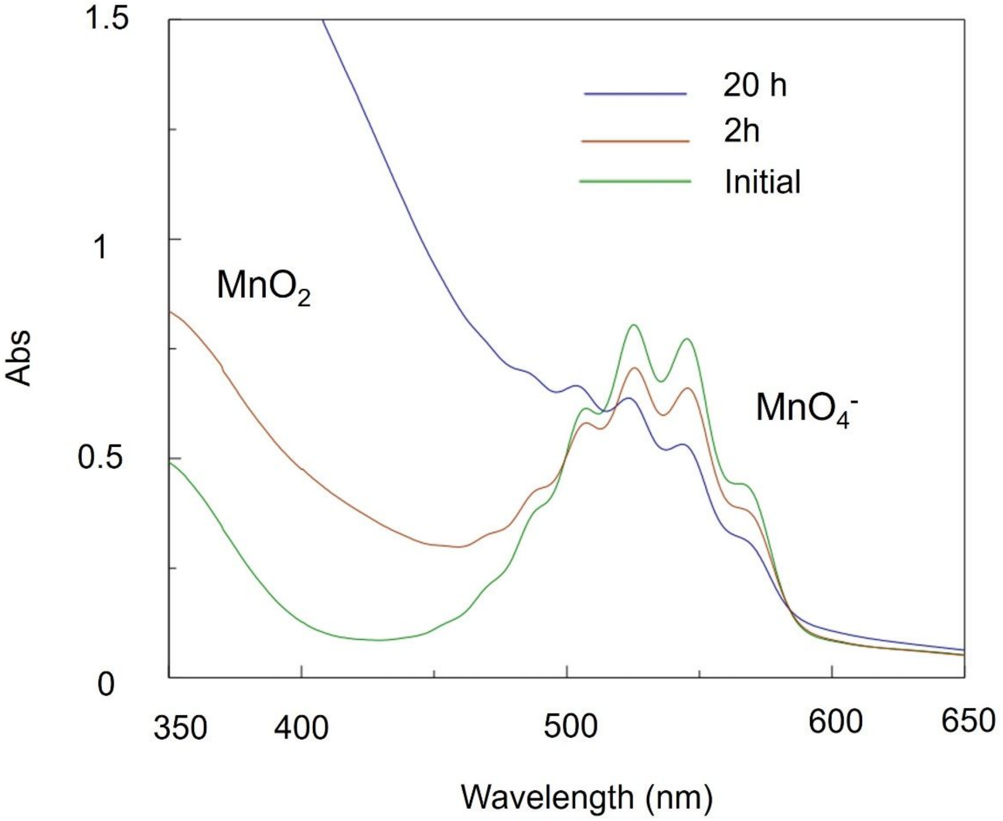

Fig. S6. Synthesis profile for **GTL@Mn2000eq**.

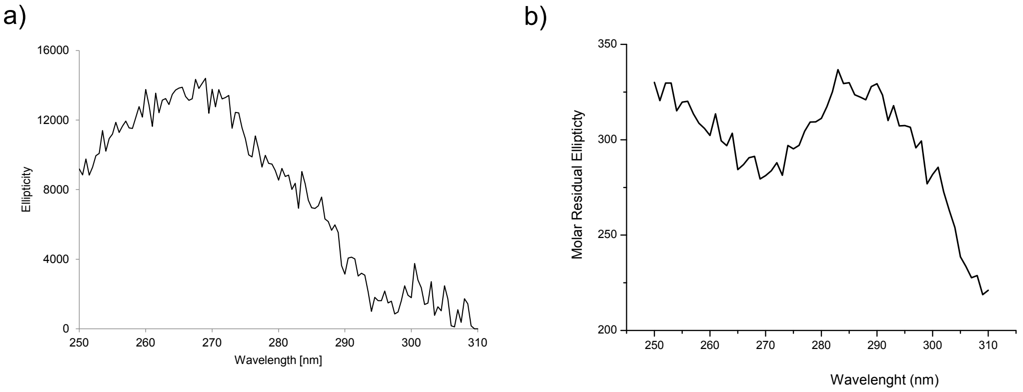

Fig. S7. Near-UV circular dichroism images. A) native GTL. B) GTL-Mn metalloenzyme.

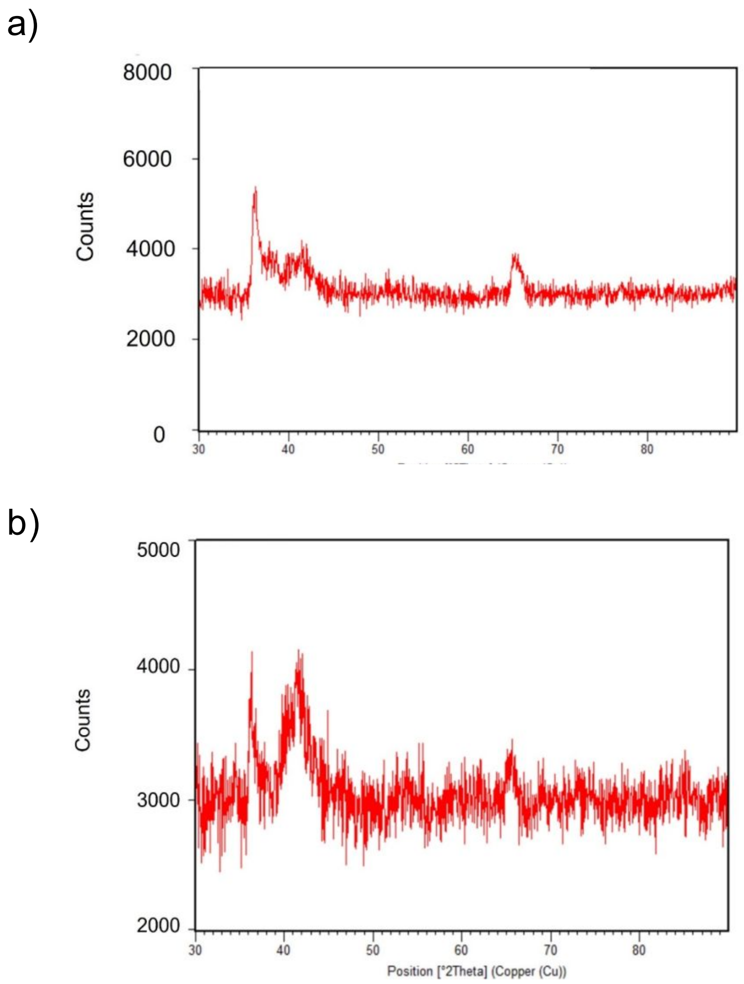

Fig. S8. A) XRD pattern for **GTL@Mn500eq**. B) XRD pattern for **GTL@Mn200eq**.

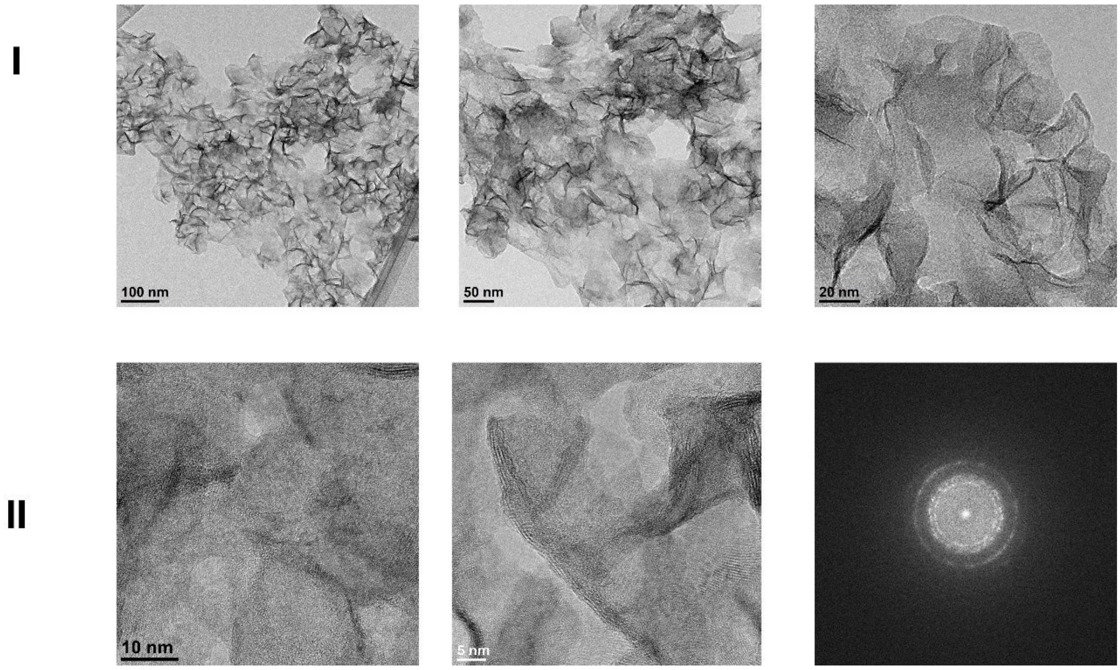

Fig. S9. Characterisation of **GTL@Mn2000eq.** **(I)** Transmission electron microscopy (TEM). **(II)** High Resolution TEM (HR-TEM) inset Fast Fourier transform (FFT) patterns from HRTEM images.

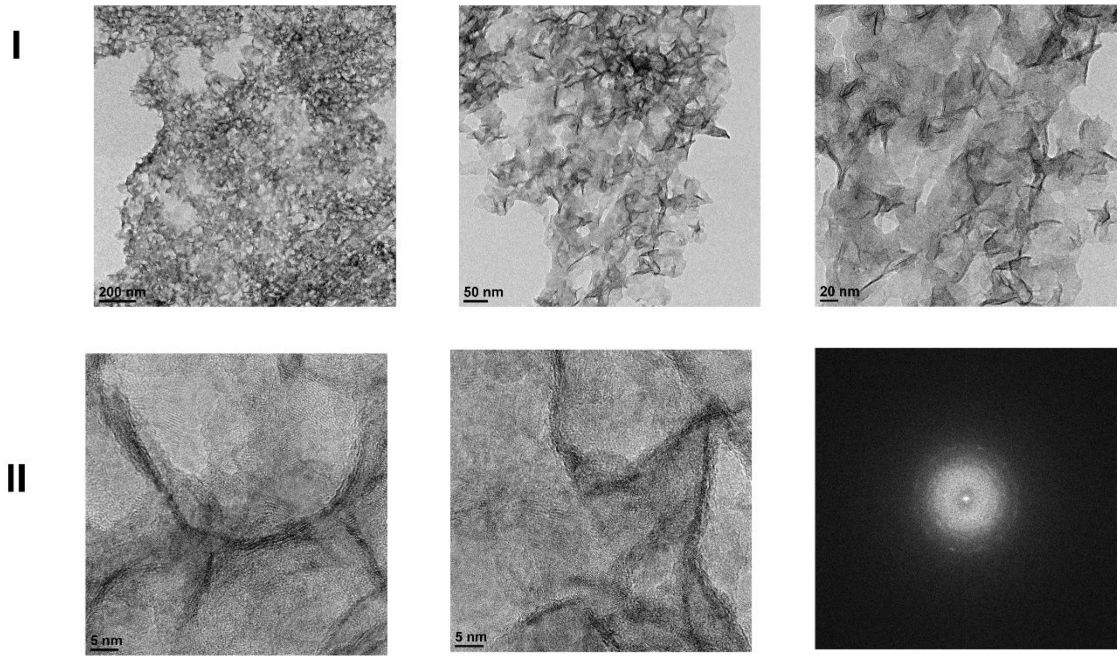

Fig. S10. Characterisation of **GTL@Mn500eq**. **(I)** Transmission electron microscopy (TEM). **(II)** High Resolution TEM (HR-TEM) inset Fast Fourier transform (FFT) patterns from HRTEM images.

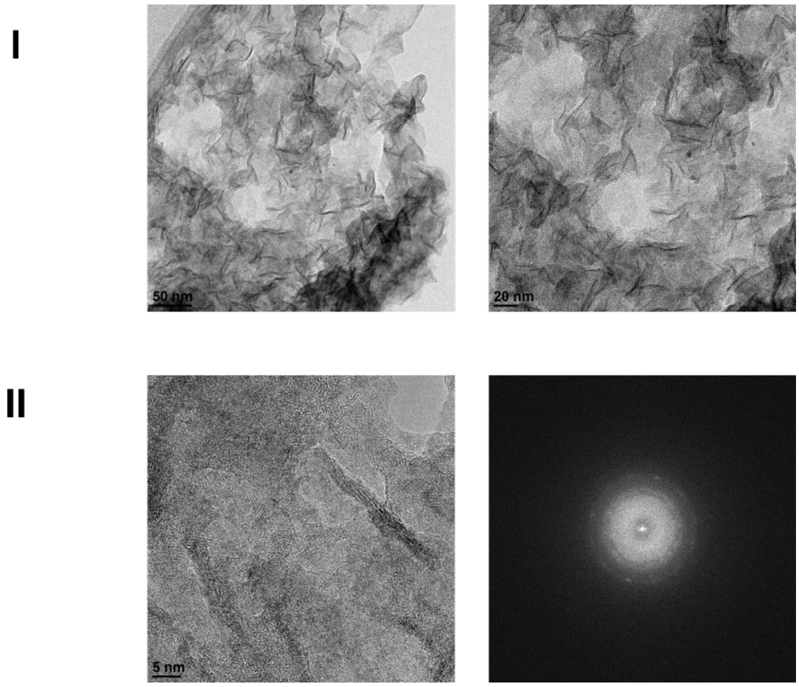

Fig. S11. Characterisation of **GTL@Mn200eq. (I)** Transmission electron microscopy (TEM). **(II)** High Resolution TEM (HR-TEM) inset Fast Fourier transform (FFT) patterns from HRTEM images.

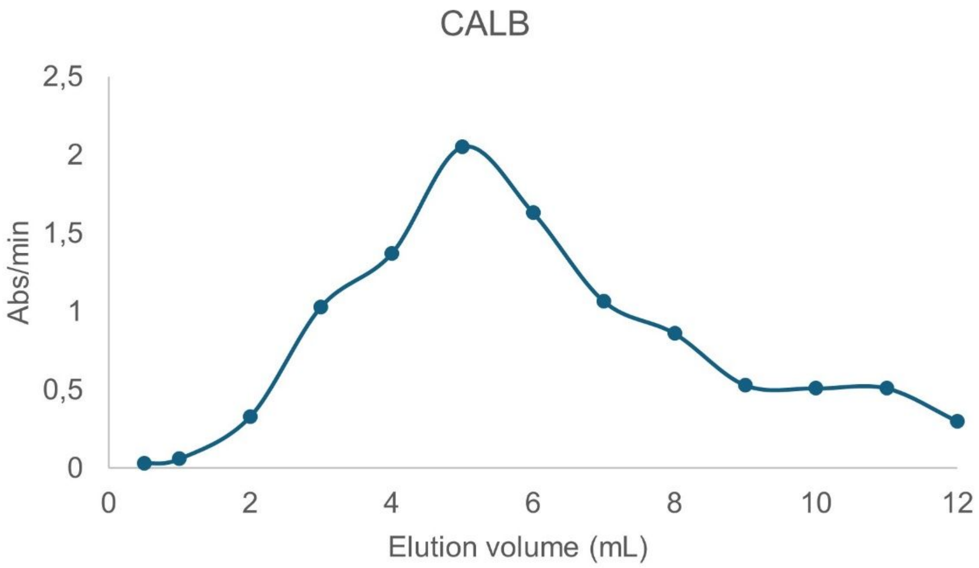

Fig. S12. Elution profile in gel filtration chromatography of CALB (33KDa).

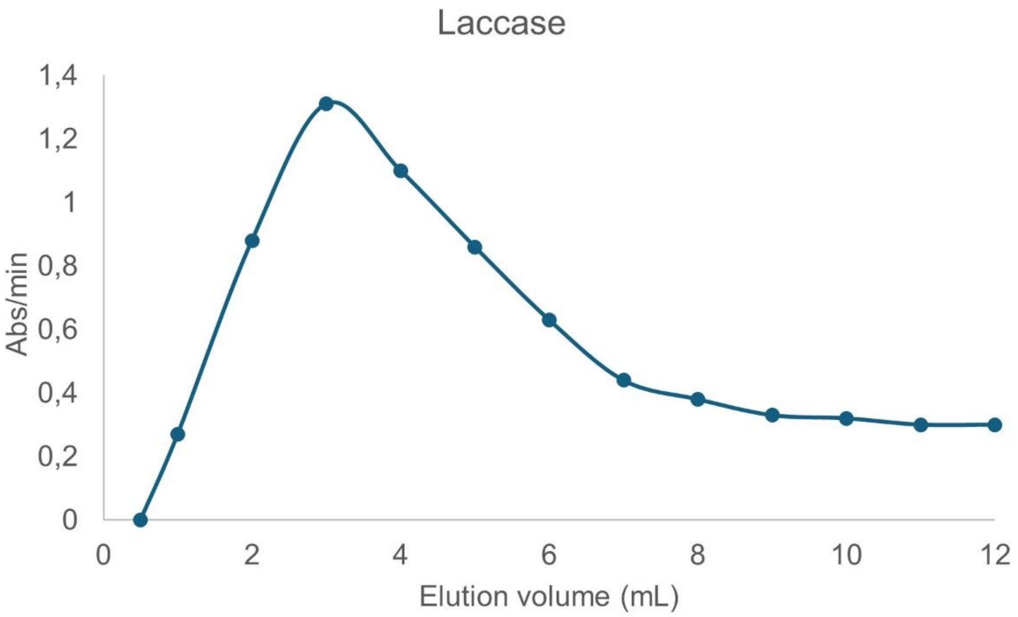

Fig. S13. Elution profile in gel filtration chromatography of laccase from *Myceliophthora thermophila* expressed in *Aspergillus oryzae* (Novozym 51003®) (85KDa).

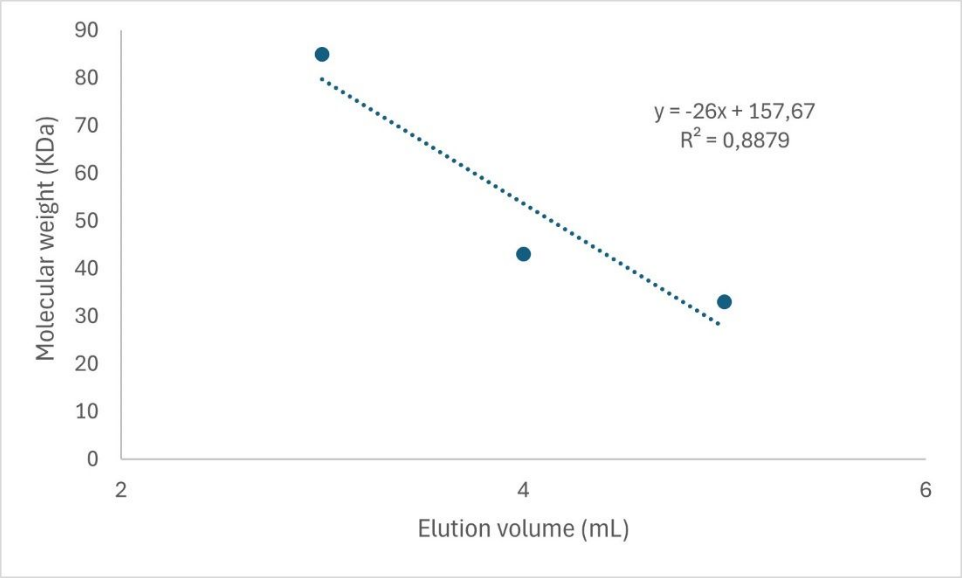

Fig. S14. Gel filtration chromatography calibration curve plotted using standard proteins (CALB, GTL and laccase from *Myceliophthora thermophila* expressed in *Aspergillus oryzae* (Novozym 51003®).

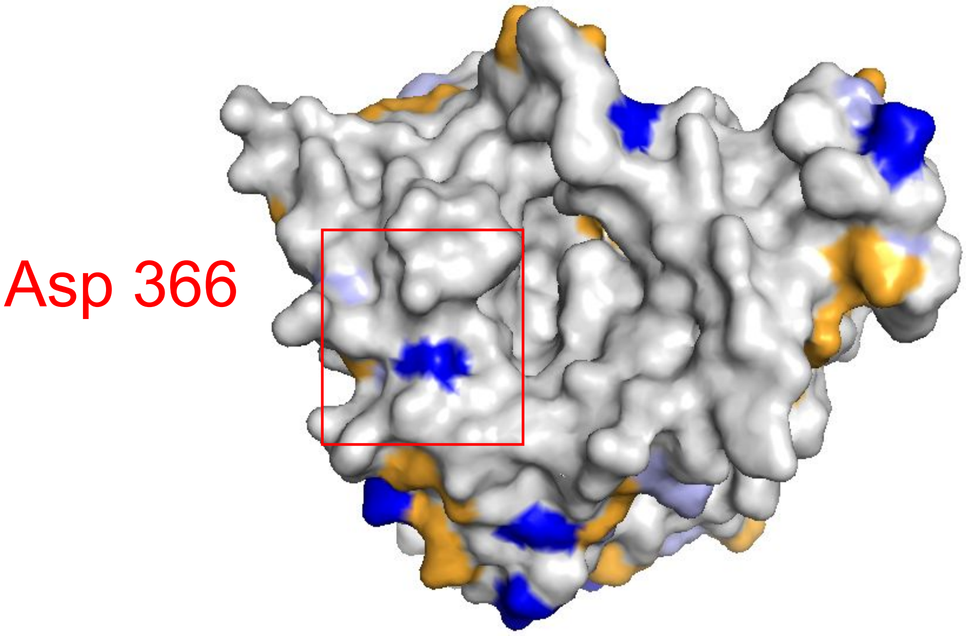

Fig. S15. Surface structure of the active conformation of GTL, marked in blue aspartic acid, in light blue glutamic acid and in orange arginine residues. The protein structure was obtained from the Protein Data Bank (PDB code: 2W22) and the picture was created using Pymol.

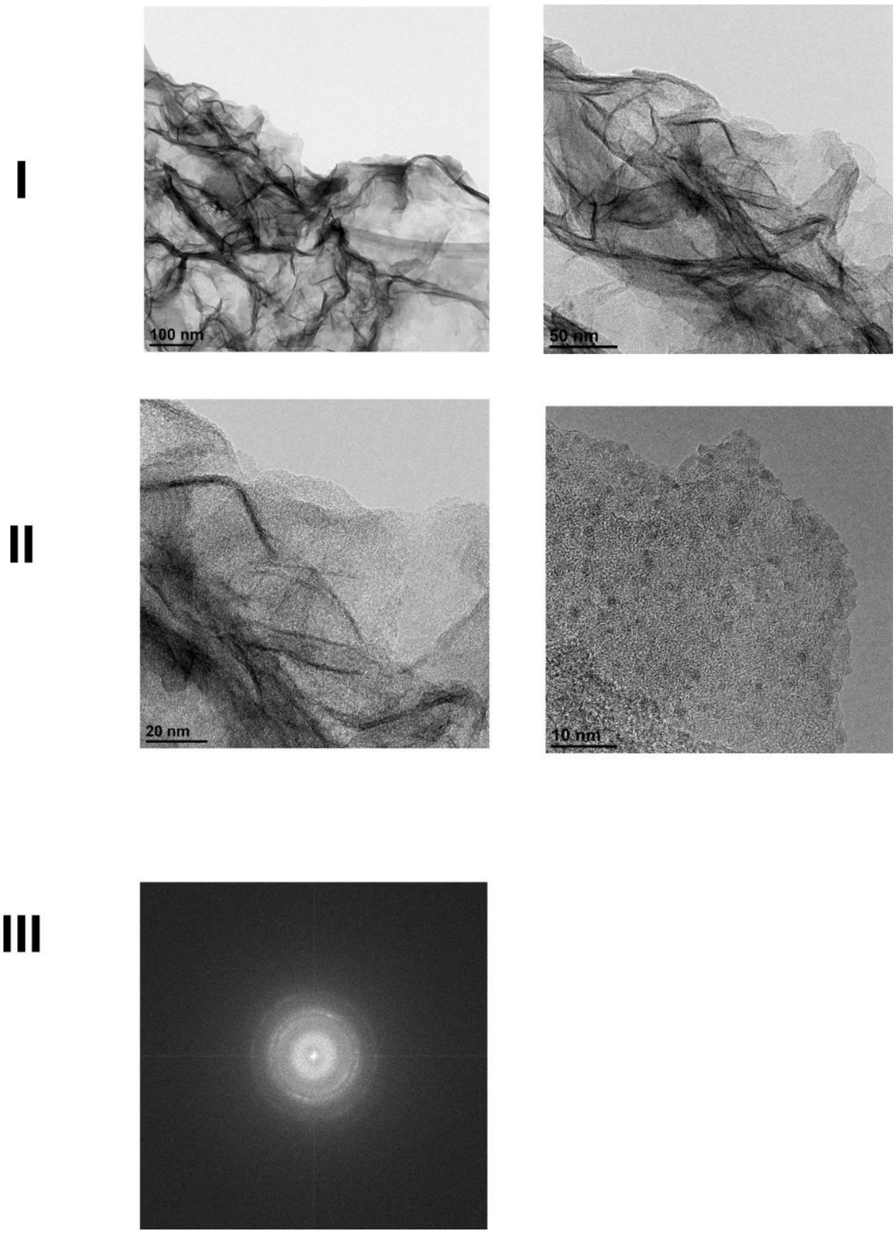

Fig. S16. Characterisation of **BuGTL@Mn2000eq.** **(I)** Transmission electron microscopy (TEM). **(II)** High Resolution TEM (HR-TEM). **(III)** Fast Fourier transform (FFT) patterns from HRTEM images.

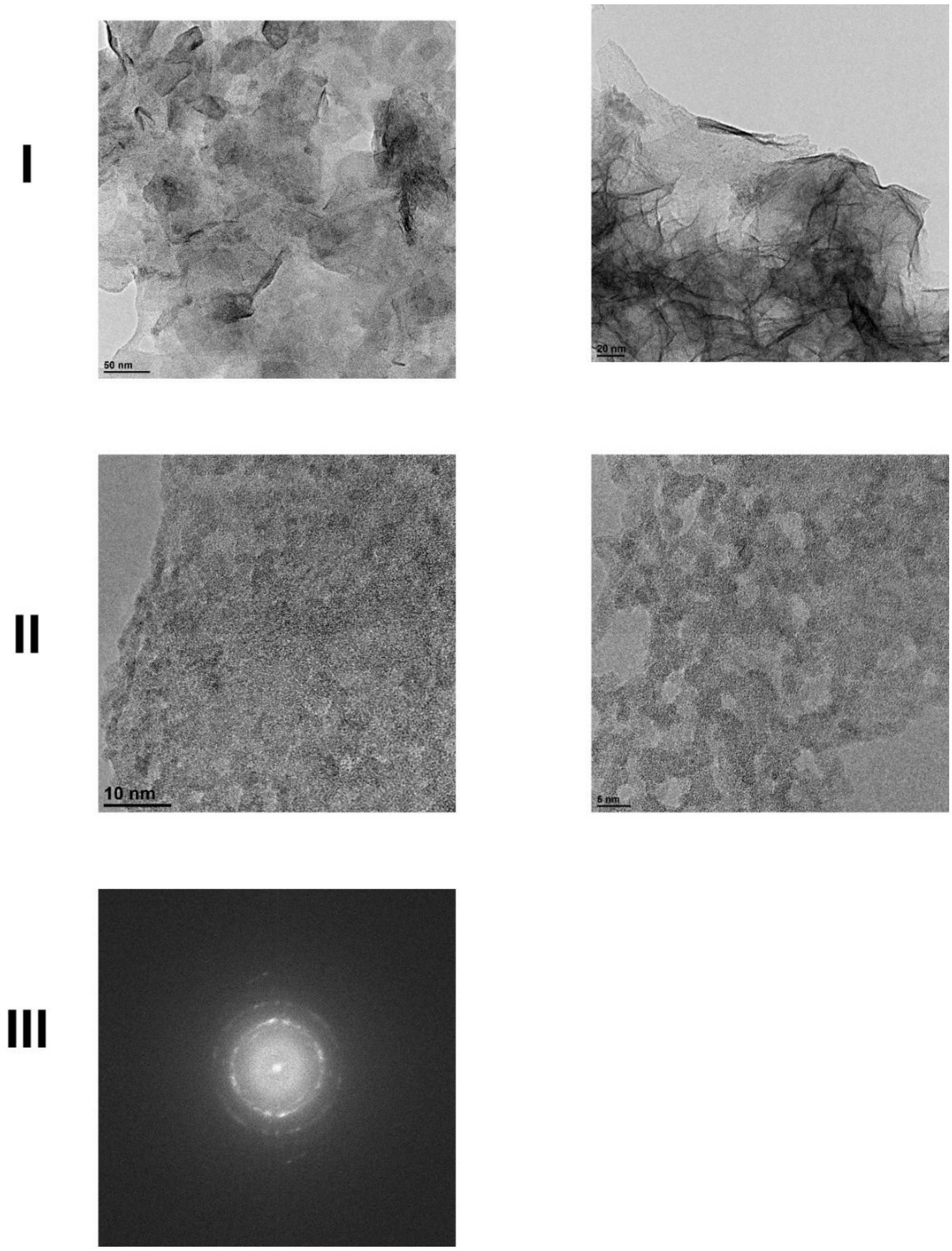

Fig. S17. Characterisation of **BuGTL@Mn200eq. (I)** Transmission electron microscopy (TEM). **(II)** High Resolution TEM (HR-TEM). **(III)** Fast Fourier transform (FFT) patterns from HRTEM images.

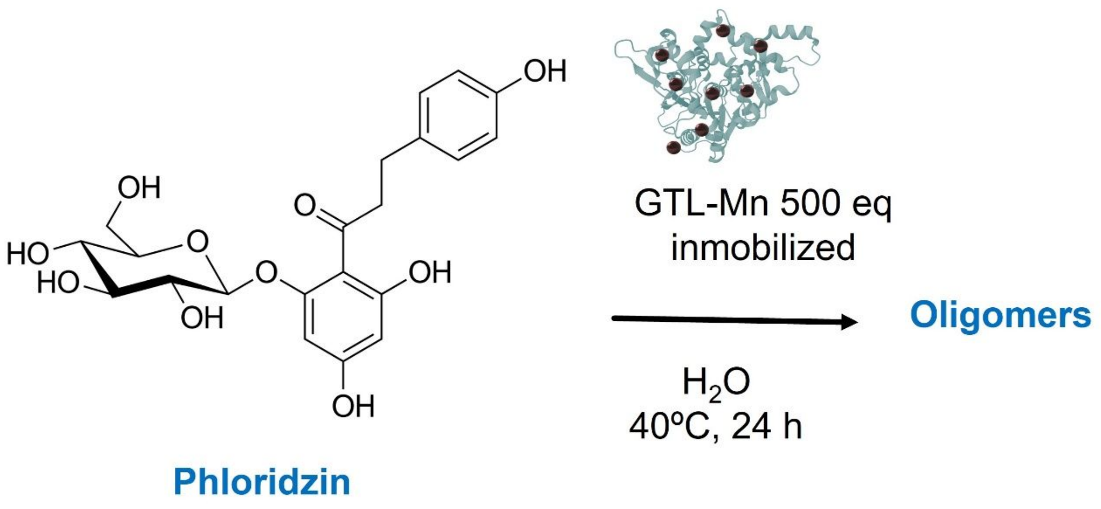

Fig. S18. Phloridzin oligomerization reaction.

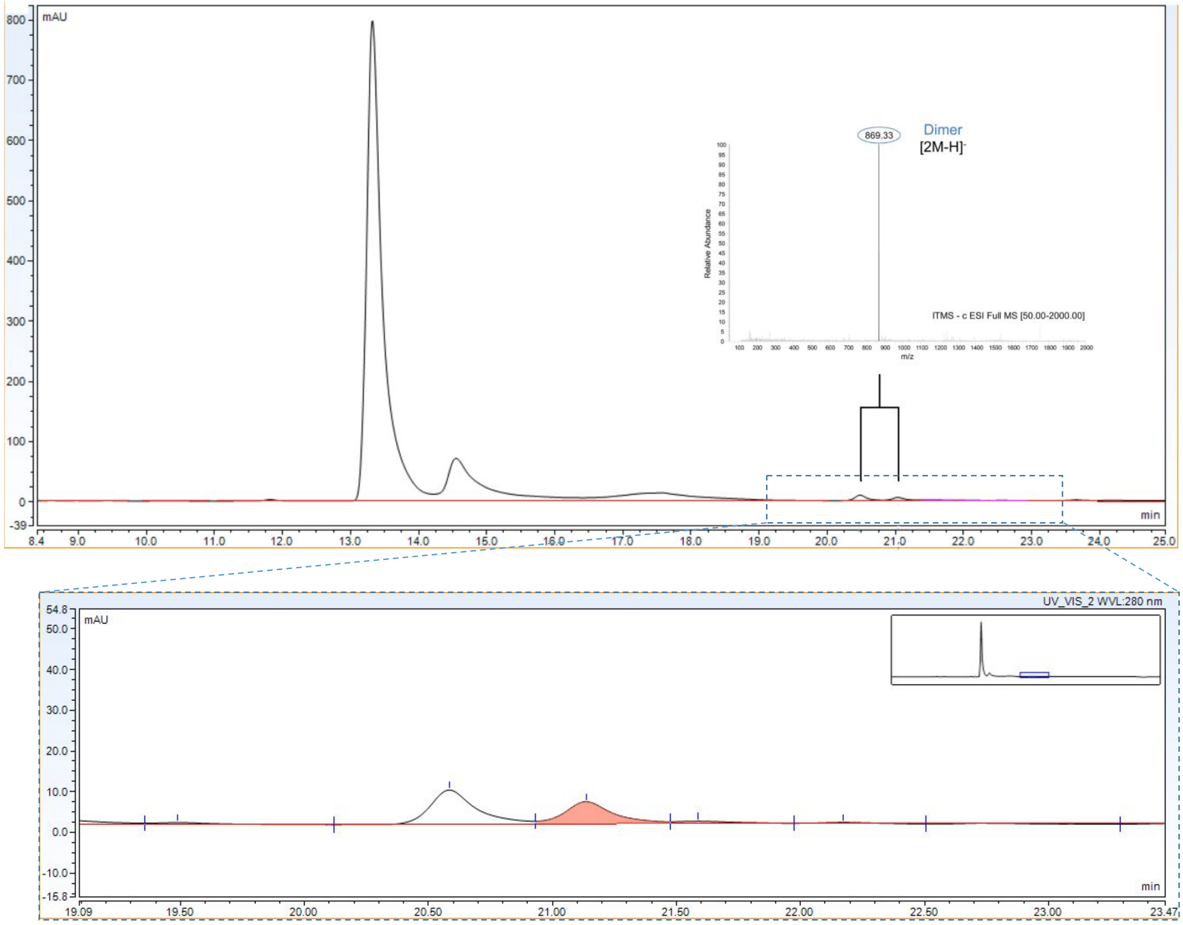

Fig. S19. Mass chromatogram of the reaction mixture after 24 h of the reaction.

Table S1. Fluorescence data for the manganese metalloenzymes.

Entry	Sample	λ emission (nm)
**1**	**Free GTL**	303
**2**	**GTL@Mn2000eq**	301
**3**	**GTL@Mn500eq**	301
**4**	**GTL@Mn200eq**	301

Table S2. Protein and Mn content of the manganese metalloenzymes.

Entry	Sample	Protein (nmol)	Mn (nmol)
**1**	**GTL@Mn2000eq**	0.16	55.1
**2**	**GTL@Mn500eq**	0.33	36.8
**3**	**GTL@Mn200eq**	0.65	19.3
